# Modeling Measurement as a Sequential Process: Autoregressive Confirmatory Factor Analysis (AR-CFA)

**DOI:** 10.3389/fpsyg.2019.02108

**Published:** 2019-09-20

**Authors:** Ozlem Ozkok, Michael J. Zyphur, Adam P. Barsky, Max Theilacker, M. Brent Donnellan, Frederick L. Oswald

**Affiliations:** ^1^Department of Management and Marketing, The University of Melbourne, Melbourne, VIC, Australia; ^2^Department of Psychology, Michigan State University, East Lansing, MI, United States; ^3^Department of Psychological Sciences, Rice University, Houston, TX, United States

**Keywords:** confirmatory factor analysis (CFA), personality factors, auto regression (AR), structural equation modeling (SEM), autoregressive model

## Abstract

To model data from multi-item scales, many researchers default to a confirmatory factor analysis (CFA) approach that restricts cross-loadings and residual correlations to zero. This often leads to problems of measurement-model misfit while also ignoring theoretically relevant alternatives. Existing research mostly offers solutions by relaxing assumptions about cross-loadings and allowing residual correlations. However, such approaches are critiqued as being weak on theory and/or indicative of problematic measurement scales. We offer a theoretically-grounded alternative to modeling survey data called an autoregressive confirmatory factor analysis (AR-CFA), which is motivated by recognizing that responding to survey items is a sequential process that may create temporal dependencies among scale items. We compare an AR-CFA to other common approaches using a sample of 8,569 people measured along five common personality factors, showing how the AR-CFA can improve model fit and offer evidence of increased construct validity. We then introduce methods for testing AR-CFA hypotheses, including cross-level moderation effects using latent interactions among stable factors and time-varying residuals. We recommend considering the AR-CFA as a useful complement to other existing approaches and treat AR-CFA limitations.

## Introduction

When people respond to multi-item scales, item responses may depend on one another due to the order in which items are presented (as suggested by Schwarz and Clore, 1983; Schwarz, 1999, 2011). In this paper we propose that ignoring these sequential, autoregressive effects may lead researchers to draw erroneous conclusions from their data. Although longitudinal and panel data literatures treat autoregressive effects at length (Arellano, 2003; Baltagi, 2013; Hamaker et al., 2015), most survey research overlooks the sequential nature of multi-item scales (Knowles, 1988; Knowles et al., 1992; Knowles and Byers, 1996). To tackle this problem, we offer a new way to model multi-item scales which present items in the same order to all respondents: an autoregressive confirmatory factor analysis (AR-CFA).

We first motivate our work by treating issues in measurement model specification and fit, including ways to improve fit such as exploratory structural equation models (ESEM) and Bayesian approaches (e.g., Asparouhov and Muthén, 2009; Marsh et al., 2009, 2010; Muthén and Asparouhov, 2012). We then explore measurement as a sequential process, describing serial dependencies as “context effects” that may emanate from two sources: (1) memory activation and (2) affective priming, both of which may cause autoregressive effects among survey items (Knowles, 1988; Tourangeau and Rasinski, 1988; Tourangeau et al., 1989; Hamilton and Shuminsky, 1990; Steinberg, 1994; Krosnick, 1999; Schwarz, 2011; Mikels and Reuter-Lorenz, 2019). We then propose the AR-CFA as a method for including AR effects in a measurement model, including latent interactions among latent factors and residuals to test “cross-level” longitudinal/panel moderation. Next, we illustrate an AR-CFA in a sample of 8,569 people responding to a Big Five personality survey, showing improved fit using an AR-CFA and hypothesis testing procedures—all data and results are available as online appendices. We conclude by emphasizing the value of theory-based measurement models, limits of our approach, and future research directions.

## Measurement Models and Their Statistical Fit

To make substantive inferences using data from multi-item scales, their psychometric properties are often studied to provide evidence of sound measurement. The most common methods involve SEMs that allow separately evaluating measurement and structural models (Williams et al., 2009). Measurement models define the relationships among observed and latent variables, as well as residual (co)variances, whereas structural models reflect hypothesized substantive relationships among latent variables (Bollen, 1989). To meaningfully interpret structural model parameters, measurement models must first pass a hurdle of satisfactory fit—typically tested by CFA—before tests for structural relationships are undertaken (Anderson and Gerbing, 1982; Mulaik and Millsap, 2000).

In practice, good measurement model fit is not always obtainable even for common scales such as personality inventories (Church and Burke, 1994; McCrae et al., 2008; Hopwood and Donnellan, 2010; Marsh et al., 2010). The problem of misfit often arises due to a desire for a classic factorial “simple structure” that was first proposed by Thurstone to evaluate a scale's characteristics using exploratory factor analysis (EFA; Thurstone, 1935, 1947). Thurstone proposed that if a scale measures underlying constructs well, then an EFA should show large hypothesized factor loadings and small or near-zero loadings elsewhere. Although this approach uses EFA in a confirmatory fashion by imposing limits on allowable cross-loadings, EFA specifies that all variables may freely load on all factors, and thus the question of model fit was not relevant for Thurstone in the same way it is today with CFA.

**Figure 1:**
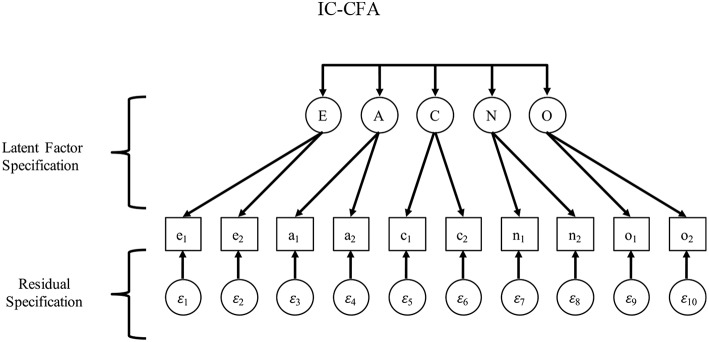
IC-CFA.

The typical CFA that has evolved since Thurstone is often called an “independent clusters” CFA (IC-CFA), which is stricter than EFA because it omits unhypothesized cross-loadings (see Figure 1; McDonald, 1985; Thompson, 2000, 2004). Today, the IC-CFA is often used in published CFAs and tends to inform researchers' and reviewers' expectations of what measurement models should look like (Asparouhov et al., 2015). The strictness of the IC-CFA model, however, causes a problem for researchers who find poor measurement model fit and then conclude that they cannot proceed to investigating structural relationships. Therefore, it is unsurprising that many scholars who assess constructs such as personality traits prefer EFA over CFA due to the commonly observed lack of good model fit in an IC-CFA (McCrae et al., 1996; Marsh et al., 2010).

The question of how to address this misfit has generated substantial debate, leading to many alternatives to the IC-CFA including exploratory structural equation modeling (ESEM) and Bayesian approaches (e.g., Marsh et al., 2010; Muthén and Asparouhov, 2012). These involve allowing for non-zero cross-loadings (i.e., items “belong” to multiple factors as in ESEM) and/or residual covariances (i.e., item errors are allowed to correlate as in Bayes methods; Asparouhov et al., 2015). Yet, owing to the idealization of an IC-CFA model and a desire to avoid inferential errors caused by model overfitting (i.e., “capitalizing on chance” in the form of sampling error variance), cross-loadings and residual covariances are often viewed skeptically (e.g., Stromeyer et al., 2015).

Certainly, when measurement model misfit is found with an initial IC-CFA, using model modification indices provided by a software package will improve model fit. Because these are usually *post-hoc* modifications based on the same sample on which the model was originally tested, it is unclear how much of the modification is due to “real” improvement that would generalize to new data vs. how much is due to overfitting and would thus vanish in new data (MacCallum, 1986; Cudeck and Henly, 1991; MacCallum et al., 1992; Chin, 1998; Williams et al., 2009). In addition to this challenge, even if cross-loadings and residual covariances are specified *a priori* (without using model modification indices), this approach is still often interpreted as a sign of a poorly functioning measurement scale because such specifications deviate from the traditional IC-CFA wherein items are “pure” indicators of one factor. Indeed, in a model with cross-loadings and residual covariances, some researchers may question the substantive meaning of structural effects and the nature of the items and scales themselves (e.g., MacCallum et al., 2012; Rindskopf, 2012).

A recent exchange in the *Journal of Management* highlights these issues. Stromeyer and colleagues note that specifying cross-loadings and residual covariances potentially allows researchers to justify weak scales and poorly specified models that can limit generalizability (Stromeyer et al., 2015). The authors suggest that cross-loadings and residual covariances may be useful as diagnostic tools, but they should not be used to improve model fit to justify interpreting structural relationships. In reply, Asparouhov et al. (2015) note that there can be good theoretical reasons to appreciate cross-loadings and residual covariances. For example, allowing errors to correlate may be reasonable because scales often have items with similar wordings and may reference similar contexts. To the extent that items do not appear to function ideally in terms of an IC-CFA structure (i.e., they do not reflect “pure” constructs with errors independent of one another), these authors note that the problem may be the IC-CFA, which is too strict and will often be less useful than a model that allows cross-loadings and residual covariances with “small-variance” priors using a Bayes estimator (which serves to “shrink” estimates of cross-loadings and residual covariances toward zero).

Because the view expressed by Stromeyer et al. (2015) is common among reviewers, researchers may tend to avoid using specifications that deviate from an IC-CFA, even though the logic of Muthén and Asparouhov (2012) and Asparouhov et al. (2015) has merit. In our view, the IC-CFA is problematic if it leads to underspecified measurement models that affect structural parameters (see Beauducel and Wittmann, 2005; Cole et al., 2007; Schmitt and Sass, 2011). More generally, the IC-CFA is problematic because there are many cases wherein deviations from it are warranted yet go unspecified due to the belief that an IC-CFA is the only desirable or defensible measurement model (Cole et al., 2007). Although one way to improve fit under an IC-CFA is to remove items that have cross-loadings and correlated errors (or develop items that lack these properties, doing this may be unreasonable or impossible in practice or otherwise be problematic.

In sum, the IC-CFA evolved from Thurstone's thinking about measurement using EFA (Thompson, 2000, 2004). Unfortunately, the IC-CFA is now too often used as a heuristic for evaluating not only the scale being subjected to a CFA, but also the type of CFA specification itself. Two key effects of this established view are: (1) Thurstone's heuristic for judging the quality of a scale based on a liberal, yet confirmatory, EFA model evolved into a very conservative IC-CFA that often ensures model misfit; and (2) by usually defaulting to an IC-CFA specification, researchers and reviewers rarely engage in theorizing about processes that may produce justifiable deviations from the expectations that an IC-CFA imposes on a dataset. In the next section, we theorize one such deviation from the IC-CFA in a justifiable and testable way by exploring the sequential process of responding to items in a scale.

## Measurement as a Sequential Process: The AR-CFA

We now offer theoretical and model-based ways to understand sequential “context effects” among survey items—although we discuss this in relation to a fixed item order across all survey respondents, we later describe how the AR-CFA can also be applied to surveys with randomized item orderings to capture respondent-specific context effects. As in classic panel data literature, we call these autoregressive or AR effects, which are effects of the past on the future. We begin with a theoretical description of the basis for these effects as follows.

### Survey Responses and Context Effects

To understand the problematic nature of defaulting to an IC-CFA, it is important to understand why one may expect typical deviations from the strict assumptions of IC-CFAs. To begin, consider the common situation that occurs when organizational researchers collect survey data (e.g., job attitudes, personality). By answering items sequentially, respondents must: (1) interpret the meaning of an item; (2) retrieve relevant beliefs, feelings, or memories; (3) apply these to the item; and then (4) select a suitable response (Tourangeau and Rasinski, 1988; Krosnick, 1999; Schwarz, 2007). Whereas, the IC-CFA treats each response as being driven by a single construct (and a residual error), each step of the response process may be affected by exposure to earlier material in the survey—often called “context effects” (see Tourangeau and Rasinski, 1988)—which can create systematic and possibly even predicable deviations in future item responses that are unaccounted for in the traditional IC-CFA.

A substantial amount of research by social and experimental psychologists supports the existence of such context effects, including work on the automatic and interdependent natures of emotions and cognition (see Cacioppo and Gardner, 1999; Forgas, 2001; Evans, 2008; Wyer, 2014), as well as the logic of experimentation with repeated-measures designs (see Shadish et al., 2002). Indeed, building on relevant social and experimental studies, we propose that various kinds of order effects *should* exist due to the fact that people must sequentially read and respond to survey items (Krosnick, 1991, 1999). For instance, studies show that item factor loadings increase as individuals move from the beginning to the end of a survey (Knowles, 1988; Hamilton and Shuminsky, 1990; Knowles et al., 1992; Knowles and Byers, 1996). This is an example of a context effect that operates in relation to the stable underlying construct of interest. By answering earlier questions about a construct, individuals become more consistent in their responses to later items, in part because there is greater accessibility of information related to the construct.

To address context effects, we focus on AR effects that capture temporal dependence among items due to the sequential nature of item responses. In an IC-CFA, AR effects are ignored because items are treated as independent except as they are affected by the measured latent factors of interest. However, existing literature notes that AR effects may exist due to basic psychological processes involved in responding to survey items (Tourangeau and Rasinski, 1988; Tourangeau et al., 1989). Below, we discuss two possible sources of AR effects: (1) memory activation (cognitive) and (2) affective priming (affective). We also treat a potential moderator of AR effects: the latent factor neuroticism. By proposing these effects, we do not mean to exclude other potential sources of AR effects or moderators, and instead simply intend to offer a starting point for understanding AR effects in survey data.

#### Memory Activation

Consider the step in which survey respondents must reflectively search their long-term memory for events or conceptual structures (e.g., attitudes or self-concepts) to answer a question. Memory retrieval works through the spreading activation of nodes (e.g., thoughts, ideas, and feelings) that are linked through an associative network. Once activated, these nodes are primed for further activation, thereby making them more likely to be used for subsequent retrieval. For example, items assessing the same construct deal with similar beliefs and behaviors, and thus retrieving information to respond to similar questions will sequentially rely on access to the same areas in memory (Knowles, 1988; Hamilton and Shuminsky, 1990; Steinberg, 1994). Therefore, blocking items together such that respondents answer several items measuring the same construct sequentially should enable a respondent to respond more accurately, partly because they expend fewer cognitive resources in the retrieval process—this helps explain larger factor loadings later in a scale (Knowles et al., 1992).

However, once a node is activated, the process of spreading activation continues automatically, potentially activating nodes that are irrelevant to the task at hand (Posner, 1978). For instance, a respondent answering the extraversion item “Don't talk a lot” may recall situations and self-concept structures consistent with social isolation or disengagement (rather than merely extraversion), which may then activate memories and attitudes that bias responses to the next agreeableness question “Am not interested in other people's problems” (these items appear in this order within the personality scale we later examine).

In such cases, the irrelevant (i.e., non-construct-related) memories that are activated would likely carry over to subsequent questions, but degrade as new memories are retrieved with each subsequent item. As such, previous items would serve as context that inadvertently affect future items due to spreading memory activation, but such AR effects may be short-lived because each new item causes new activations that then subsequently spread. Such AR effects are theorized as being due to carry-over in node activation—a cognitive effect. These effects should be positive when activated nodes are consistent, such as the “Don't talk a lot” and “Am not interested in other people's problems” items. On the other hand, negative effects should arise when nodes are countervailing, such as if the latter item read “*Am interested* in other people's problems.” Again, such effects are distinct from the intended measurement characteristics of a scale, which include factor loadings that potentially increase over time but also latent covariances among factors due to any true-score covariance (e.g., more extraverted people may be more agreeable, as in our later example).

#### Affective Priming

Alternatively, mood states are highly susceptible to context and can be primed and then used directly as information by respondents (see Schwarz, 2011). Because survey questions can generate positive or negative affect, it follows that serial dependencies may exist that link item responses even if the affect involved is unrelated to the substantive constructs of interest. For example, the extraversion item “Don't talk a lot” could generate affect consistent with the response (i.e., those who don't talk a lot might experience negative affect by endorsing such an antisocial item). In turn, this affect could inform responses to the next question “Am not interested in other people's problems,” because it implies a kind of social disengagement that may be associated with negative affect, creating a positive association among the adjacent items even though affect itself is not being measured.

Thus, serial AR effects may exist when a previous item causes an affective reaction and responding to a future item relies on the affect generated. In such cases, the affect is likely to carry over to the following item(s) systematically, such that the affect generated by a previous item and used for a future response will cause positive AR effects if the affective implications of the items are positively linked (e.g., the extraversion and agreeableness items mentioned here), but negative AR effects may exist when items are negatively linked (e.g., if the agreeableness item read “*Am interested* in other people's problems”). Furthermore, as with memory activation effects, the AR effects induced by affective priming should fade with time as more items are responded to and previous items recede further into the past. In conclusion, the effects of affective priming typically fade over time, such that the time-varying AR effects of past responses should fade as respondents move from one item to another.

#### Cross-Level Moderation of AR Effects

If cognitive and affective processes create AR effects among item responses, it is possible that any psychological factors that affect these processes will moderate the AR effects. This is important because if such moderation occurs in a manner that is theoretically consistent with the mechanisms described above, this could serve as partial evidence that AR effects are driven at least in part by context.

To address this possibility, it is important to recognize that neuroticism has been positively associated with both high levels of rumination (e.g., a cognitive AR mechanism) as well as greater emotional reactivity to stimuli (e.g., the affective AR mechanism). Indeed, literature on affective inertia shows people high in neuroticism tend to have larger AR effects that reflect the persistence of emotions over time (Suls et al., 1998; Koval et al., 2016). These AR effects are driven by a tendency to ruminate by keeping past events or scenarios in mind (Koval et al., 2012), rather than updating these in working memory on a moment-to-moment basis (Pe et al., 2013). Thus, if AR effects are driven by memory activation and affective priming, we would expect individuals higher in neuroticism to have larger AR effects, whereas those lower in neuroticism would have smaller AR effects (i.e., AR effects closer to zero).

#### Summary and Hypotheses

In sum, the memory activation and affective priming that occurs when individuals read and respond to survey items (i.e., context effects) allows us to theorize that: (1) AR effects may exist between items measuring the same construct (what we will term “within-construct” effects) even if dispersed in a survey; (2) AR effects may exist between items of different constructs (what we will term “between-construct” effects) when these items are adjacent; and (3) neuroticism will moderate AR effects, such that higher neuroticism will be associated with larger AR effects. Certainly, we could draw on additional theory to justify potential sources of context effects, such as anchoring (for insight see Epley and Gilovich, 2001, 2006). However, the purpose of our paper is to demonstrate that there may be consistent responding in survey data for reasons that are unrelated to standings along the underlying constructs being measured, thus systematically violating an IC-CFA structure in a theoretically meaningful way. To show how to incorporate our theorized AR effects into CFAs, we now describe our proposed AR-CFA model.

### Introducing the AR-CFA

To introduce the AR-CFA, we first show how the IC-CFA ignores sequential AR effects because it is grounded in an assumption that the process of responding to items causes dependence among items only due to the common construct(s) referenced in a set of items. This assumption can be formalized as an IC-CFA as follows (see Figure 1):

(1) $${\text{y}}_{\text{i}}^{*}=\nu^{*}+\Lambda^{*}\eta_{\text{i}}^{*}+\varepsilon_{\text{i}}^{*}$$

with a superscript ^*^ distinguishing these terms from those in subsequent models; ${\text{y}}_{\text{i}}^{*}$ is a *p* × 1 vector of *p* observed items for person *i*; ν^*^ is *p* × 1 vector of item intercepts; Λ^*^ is a *p* × *m* matrix of factor loadings that *uniquely* link each observed item to a single latent variable *m* = 1, 2, … *m* (i.e., no cross-loadings, meaning only one non-zero loading for each of the *p* rows of Λ^*^); $\eta_{\text{i}}^{*}$ is an *m* × 1 vector of standings on *m* latent variables for person *i*, distributed as $\eta_{\text{i}}^{*}$ ~ *MVN*(α^*^,Ψ^*^), where α^*^ is an *m* × 1 vector of latent variable means (typically restricted to zero because this helps identify the model, and latent means are usually arbitrary); Ψ^*^ is a *m* × *m* symmetric matrix of unrestricted latent variable variances and covariances; and, $\varepsilon_{\text{i}}^{*}$ is a *p* × 1 vector of latent disturbances for each person *i*, distributed as $\varepsilon_{\text{i}}^{*}$ ~ *MVN*(0, Θ^*^), wherein Θ^*^ is an *m* × *m* diagonal matrix of residual variances (i.e., no residual covariances).

As with any measurement model, observed scores are specified as being determined by latent factors and disturbances (see Bollen, 2002), which is imposed by restricting cross-loadings and residual covariances to zero. If the IC-CFA fits a set of survey data well, then the covariance among *p* observed variables can be thought of as entirely caused by (or arising from) the *m* latent variables (including their latent covariances), wherein *m* is typically much smaller than *p* (i.e., the factor model is parsimonious, with fewer factors than items).

Clearly, the IC-CFA specification makes a number of very strong assumptions. As noted previously on a more conceptual level, there is no allowance for items to reference multiple latent variables (which could justify cross-loadings in Λ^*^) or for similar wordings or contexts to be referenced across items (which could justify covariances in Θ^*^; Cole et al., 2007; Marsh et al., 2010; Muthén and Asparouhov, 2012; Asparouhov et al., 2015). Although both situations may be plausible, we have already mentioned that the criticism of arbitrariness has been raised (e.g., Stromeyer et al., 2015). To address this issue, we propose that AR context effects can be expected to exist in survey data, implying that measurement model misfit may be at least partially due to theoretically-reasonable AR context effects.

**Figure 2:**
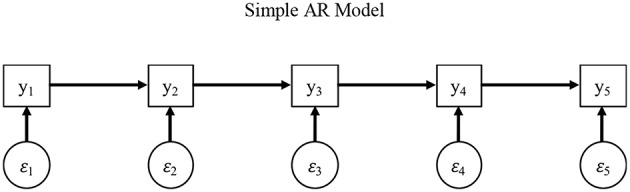
Simple AR model.

To test such AR hypotheses and demonstrate AR dependency, we begin by illustrating a model wherein these context effects are evidenced by scores along a current item *y*_*i, p*_ depending to some degree on a previous item *y*_*i, p* − 1_. We show such sequential dependence as follows (for a more general treatment, see (Hoyle, 2012; Baltagi, 2013):

(2) $$y_{\text{i},p}=\nu_{p}^{\cdot }+\kappa_{p,p-h}^{\cdot }y_{\text{i},p-h}+\varepsilon_{\text{i},p}^{\cdot }$$

wherein we use the superscript ^∙^ to distinguish these terms from those in subsequent models, and all terms are as before except the *i*th individual's score on the *p*th item *y*_*i, p*_ depends on the previous item *y*_*i, p*−*h*_ with a lag *h* = 1, so that the AR term $K_{p,p-h}^{*}$ captures the dependence of an item *p* on a previous item *p* − *h* (i.e., $K_{p,p-h}^{*}$ is an AR effect). Consistent with Equation (1), a more general AR representation can be shown in matrix form as follows (see Figure 2):

(3) $${\text{y}}_{\text{i}}=\nu^{\cdot }+\kappa^{\cdot }{\text{y}}_{\text{i}}+\varepsilon_{\text{i}}^{\cdot }$$

wherein all terms are as before except *K*^•^ is a *p* × *p* matrix of AR regression coefficients, including all AR terms *K*^•^ that link item responses in a scale over time.

As we note later, the AR terms in *K*^•^ may not capture all forms of potential temporal dependence such as respondent fatigue, but these AR terms can capture memory activation and affective priming as previous item responses affect future responses. If such effects exist, it follows that the typical IC-CFA in Equation (1) can be extended by the logic of an AR model in Equation (3), justified by the idea that item responses can reflect the effects Λ^*^ of latent variables $\eta_{\text{i}}^{*}$ as well as AR effects *K*^•^ that link past and future items in *y*_*i*_ In turn, by estimating an IC-CFA without accounting for AR effects, estimates of latent variable (co)variances and measurement model fit may be biased on a regular basis.

Indeed, the existence of stable latent variables alongside AR effects has received substantial treatment in the longitudinal/panel data modeling literature, and many techniques have been developed to account for both (see Arellano, 2003; Baltagi, 2013; Moral-Benito, 2013). Despite this work, an IC-CFA is often specified in survey research without attempting to model the fact that item responding is a sequential process that might induce AR effects. To account for these effects, the logic of Equations (1) and (3) can be synthesized in an SEM framework (similar to Moral-Benito, 2013; Hamaker et al., 2015). Before treating this, it is important to note that the logic of a typical AR model as in Equation (3) presents some interpretational difficulties when also attempting to model stable, time-invariant latent variables as factors in an IC-CFA (Hamaker, 2005; Hamaker et al., 2015).

In brief, distinguishing the effects of latent factors vs. AR terms requires an AR model in residuals only, rather than among observed variables as in Equation (3). Theoretically, this separation is akin to proposing that the context effects due to AR terms cause variance in observed data that is conceptualized as error which is unrelated to the substantive latent variables of interest in a CFA. Theoretically speaking, this is akin to the memory activation and affective priming effects *not* being related to the latent factors typically of interest in CFAs, which themselves are theorized as causing stable (co)variance in observed variables (Bollen, 2002). This is consistent with the longitudinal/panel data literature wherein latent factors are meant to reflect stable, individual-specific variables over time (Hamaker et al., 2015), whereas AR effects indicate more transient perturbations that fade with time.

**Figure 3:**
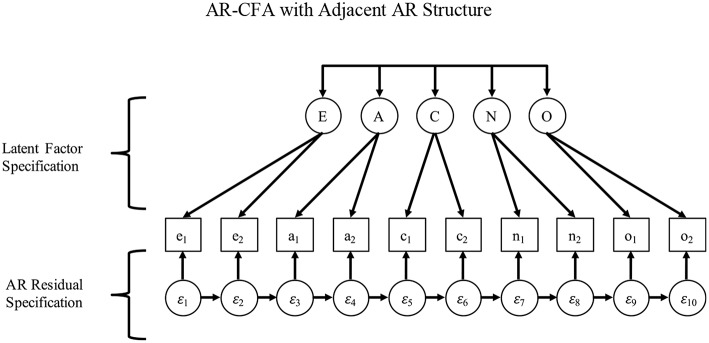
AR-CFA with adjacent AR structure.

We formalize this logic with an AR-CFA model that combines the logic of Equations (1) and (3) (see Figure 3), allowing for a typical IC-CFA specification as in Equation (1), but also specifying an AR structure to capture AR context effects in residuals as follows:

(4) $${\text{y}}_{\text{i}}=\nu +\Lambda_{\eta }\eta_{\text{i}}+\Lambda_{\varepsilon }\varepsilon_{\text{i}}$$

and

(5) $$\varepsilon_{\text{i}}=\kappa \varepsilon_{\text{i}}+{\text{u}}_{\text{i}}$$

with all terms as before except we have a typical factor loading structure for latent factors in **Λ**_**η**_ (as in an IC-CFA), which is conceptually separate from a *p* × *p* matrix **Λ**_**ε**_ with on-diagonal elements fixed to unity and off-diagonal elements fixed to zero to capture latent residuals in **ε**_**i**_, and we then show a matrix of AR effects among residuals as *K*, with ultimate residuals now captured by a *p*-length vector **u**_**i**_, where **u**_**i**_ ~ *MVN*(0, **Θ**). Also, we remove all superscripts from Equations (4) and (5) to signify that any bias induced by AR effects (omitted in Equation 1) and latent variables (omitted in Equation 3) is no longer present, with the AR-CFA allowing a traditional IC-CFA (or a different) structure for factor loadings **Λ**_**η**_ and latent covariances **Ψ**, combined with the AR effects in **κ**. Also, latent interactions among factors in **η**_**i**_ and residuals in **ε**_**i**_ can be included in the model by stacking them in the latent vector **ε**, with any resulting regression coefficients (on the interaction terms) being stacked in **Λ**_**ε**_. For brevity, we omit a technical description of this point and instead show how to construct and use the latent interactions in Mplus in our Online Appendix A and .zip file (Supplementary Material), but absolutely interested readers can examine more technical discussions of latent interactions at their discretion (e.g., Preacher et al., 2016).

The result is a model in which the covariance structure can be shown as:

(6) $$\Sigma =\Lambda_{\eta }\Psi \Lambda^{\prime }{}_{\eta }+\Lambda_{\varepsilon }{\left(\text{I}-\kappa \right)}^{-1}\Theta {(\text{I}-\kappa {)}^{\prime }}^{-1}\Lambda^{\prime }{}_{\varepsilon }$$

with all terms as before, but **Σ** is a *p* × *p* covariance matrix and **I** is an identity matrix. With this approach, unknown parameters in Equations (6) can be estimated with typical methods such as maximum likelihood (ML) or a Bayesian Markov Chain Monte Carlo (MCMC) approach—although latent interactions are only possible under ML with numerical integration. Also, by rearranging (Equation 6) as a difference equation (in Equations 7, 8), it becomes clearer how the IC-CFA factor structure is separated from the residual AR terms, which can be shown either as:

(7) $$\Sigma -\Lambda_{\eta }\Psi \Lambda^{\prime }{}_{\eta }=\Lambda_{\varepsilon }{\left(\text{I}-\kappa \right)}^{-1}\Theta {(\text{I}-\kappa {)}^{\prime }}^{-1}\Lambda^{\prime }{}_{\varepsilon }$$

or

(8) $$\Sigma -\Lambda_{\varepsilon }{\left(\text{I}-\kappa \right)}^{-1}\Theta {(\text{I}-\kappa {)}^{\prime }}^{-1}\Lambda^{\prime }{}_{\varepsilon }=\Lambda_{\eta }\Psi \Lambda^{\prime }{}_{\eta }$$

such that, in both cases, the covariances in **Σ** are essentially adjusted by either the latent factors in **η** or the residuals in **ε** to estimate the parameters associated with the other.

As Figure 3 and Equations (4)–(8) show, the AR terms in *K* have two desirable properties that are consistent with our theorizing about context effects. First, they exist in residuals and therefore are unrelated to the systematic responding associated with the substantive latent factors of interest in a typical CFA. For example, personality scale items may be ordered as Extraversion (e), Agreeableness (a), Conscientiousness (c), Neuroticism (n), and Openness (o), so that sequential AR effects would be e1 → a1 → c1 → n1 → o1 → e2 → a2 → c2 → n2 → o2. In this case, it may seem that factor loadings and latent covariances would account for the AR effects due to their repeating nature, but this is not the case because of the serial nature of the effects. For example, the similar e1 → a1 and e2 → a2 effects may appear to induce true-score covariance among extraversion and agreeableness. However, because e1 is very distant from a2, a typical CFA factor structure and latent covariances could not account for the AR effects, which would appropriately occur in the residual structure implied in Figure 3 and Equations (4)–(8).

Second, because of their serial nature, AR effects allow past context effects to fade with time, being multiplied along AR paths as indirect effects. This longitudinal relationship results from Weiner or Markov processes that induce a “simplex” pattern in correlation matrices (see classic work by Jöreskog, 1970, 1978), where correlations are stronger among closer observations (i.e., strongest near the diagonal, and fading away from the diagonal). In turn, this sets up a logical model comparison that pits the AR-CFA against a model that has equivalent *df* : a CFA that reparameterizes AR terms as residual correlations. If the context effects associated with an AR-CFA fade with time (as indirect effects along AR paths, e.g., AR effects from e1 will fade much more in item n1 compared to item a1), then the AR-CFA should provide a superior fit to the data because correlated residuals cannot capture fading correlations over time as freely-estimated indirect AR effects can. Free correlated residuals will cause model under-identification issues, and the constraints required to identify them in a way that is consistent with a freely-estimated AR structure cannot be known *a priori*.

Before showing this with a worked example, we point out that many authors note that AR specifications such as in Equations (4)–(8) can be shown as infinite-order “moving average” models (Hamaker, 2005). However, discussing this and other points related to longitudinal data models is not our goal (for this, the reader can consult general work on these models, such as Hamaker et al., 2005, 2015; Jebb and Tay, 2017).

Instead, we emphasize that, with some expected number of latent factors *m* > 1 being measured by a scale, there are multiple ways to specify the AR structure in **K**. Consistent with a classic interest in determining an AR “lag order” (i.e., the number of prior occasions on which a current observation depends; Akaike, 1969; Hannan and Quinn, 1979), an AR-CFA allows various types of AR dependence across items over time in **K**, which can be tested in various ways we discuss later. Because our purpose here is to introduce AR-CFA thinking, we initially restrict our focus to simpler lag structures such as in Figure 3, wherein item residuals depend on past item residuals, with the previous item having the strongest influence and prior items showing weaker, indirect effects along AR paths (which can be traced in Figure 3). However, with the possibility of multiple latent variables *m* > 1 and multiple ways of ordering items in a scale, there are certainly additional, theoretically reasonable AR structures that researchers might consider for specifying terms in **K**.

**Figure 4:**
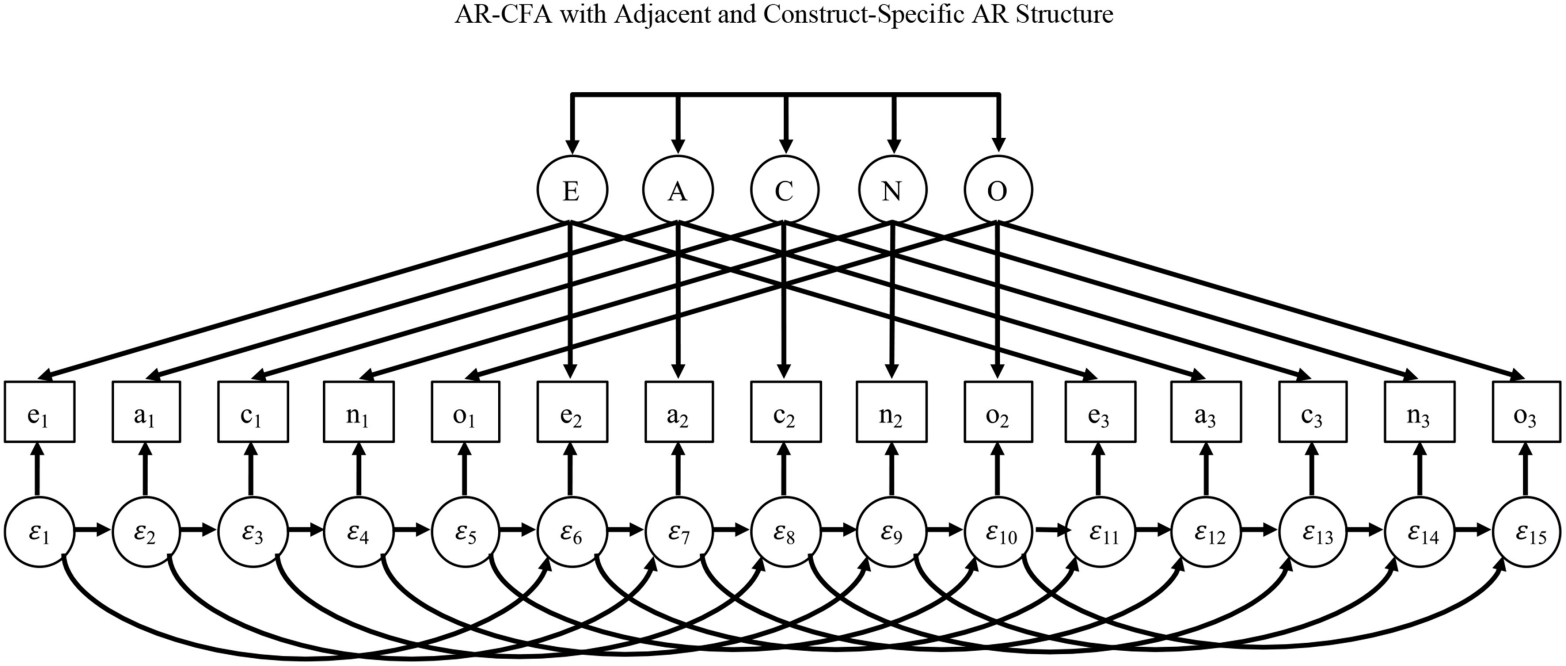
AR-CFA with adjacent and construct-specific AR structure.

To illustrate, we offer two AR structures based on two common ways of ordering scale items. Consider Figure 3, which shows items administered in a construct-oriented way, with items referencing a common construct sequentially administered in batches, producing many “within-construct” AR terms among items that measure the same construct, but “between-construct” AR terms only at the end/beginning of item sets. For this specification, a simple first-order AR model (as in Equation 2) may be appropriate to capture memory activation and affective priming that link adjacent items. To see why, consider Figure 4, which shows the common case of items distributed in a scale, such as a personality inventory, with items systematically ordered to reference different constructs that are cycled over time (e.g., Donnellan et al., 2006). In this second case, first-order dependence may exist among adjacent items, but what may also occur are higher-order dependencies among items that share a common construct, again due to memory activation and affective priming.

For example, assume a person responds to an item referencing the personality variable of extraversion, and then responds to other items referencing different personality constructs before returning to a second extraversion item. Here, the original neuroticism item may have a first-order effect on responses to the next item, but it may also affect the interpretation of (and responses to) the next extraversion item due context effects rather than the extraversion factor itself. The AR specification in Figure 4 models this with immediate AR effects among adjacent items and AR effects that link items referencing similar constructs over time (i.e., first-order between-construct AR effects and more dispersed within-construct AR effects).

There are three points to make about this second AR structure in Figure 4. First, the two types of AR effects are automatically present in Figure 3 because here first-order AR terms capture immediate dependence *and* they link adjacent items when they reference the same construct. Second, by linking items that reference the same construct, the AR structure in Figure 4 may appear to inappropriately capture covariance due to common constructs, but this is not the case because AR effects are among residuals only.

Third, the simpler AR structure in Figure 3 allows AR effects to travel in a simple indirect way across adjacent items over time, but the model in Figure 4 allows for more complex indirect relationships, with effects from the past potentially persisting along adjacent AR paths as well as those that link items reflecting similar constructs. For example, an extraversion item may cause a context effects on responses to the next item, but also a context effect on responses to the next extraversion item, and in both cases this context effect may persist beyond these two subsequent items. Because AR paths will often be rather small—as we show later—the indirect effects of *p – 2, p – 3, …, p – h* past items will quickly fall, but the AR terms still allow for data-driven lagged effects beyond the *p – 1* items, particularly in Figure 4, without needing to specify many higher-order lags.

Given the fact that many surveys separate items referencing a common construct as in Figure 4, and that the AR structure in this model is the more complex of the two we describe, we now offer an AR-CFA model implied by Figure 4. For illustration, we compare this to: (1) a traditional IC-CFA; (2) a logical comparison model with all AR terms from an AR-CFA expressed as residual correlations; (3) an EFA that mimics an ESEM with all latent variable covariances freely estimated (see Marsh et al., 2010); and (4) two Bayesian methods that improve measurement model fit using small-variance or “shrinkage” priors—one that allows all possible cross-loadings in **Λ** and one with all possible residual covariances in **Θ** (see Muthén and Asparouhov, 2012; Asparouhov et al., 2015). Elaborating on these approaches, we also estimate an AR-CFA with a Bayes estimator that sets small-variance priors for AR effects to model a case wherein AR effects are assumed to be zero. To allow comparing all models estimated, we present Bayes fit indices for all models and more common maximum-likelihood (ML) indices when this estimator can be used. Following the model comparison results, we also describe how more complex AR effects (e.g., fatigue) can be captured by imposing testable constraints on AR terms in the AR-CFA.

## Method

We illustrate and compare the above models on a short Big Five personality scale called the mini-IPIP (International Personality Item Pool), which was originally presented in Donnellan et al. (2006). This scale was developed due to practical concerns regarding the length of traditional personality inventories. Although our AR-CFA can be used on larger scales, the shorter mini-IPIP facilitates more concise tables and Mplus code while exemplifying typical dilemmas regarding measurement model fit using an IC-CFA, which is common for Big Five scales (see McCrae et al., 1996, 2008; Marsh et al., 2010).

### Sample

We illustrate and compare the AR-CFA using a dataset from the Multi-Site University Study of Identity and Culture (MUSIC) survey (see Castillo and Schwartz, 2013; Weisskirch et al., 2013; Corker et al., 2017), which collected data from 30 colleges and universities in the United States, with a sample size of *N* = 8,569 (for additional details, including relevant demographic characteristics, see Corker et al., 2017). Because this sample involves non-independence due to clustering by college/university, we group-mean centered these data around the college/university average to remove these fixed effects.

### Procedure

Students were recruited from different courses (e.g., psychology, business) by online study announcements, offering course credit for participation or entering a prize draw. Data collection was between September 2008 and October 2009, and the mini-IPIP survey took 1–2 h to complete. Corker et al. (2017) study materials are available at https://osf.io/if7ug/.

### Measures

The mini-IPIP assesses the following five personality traits in this order: Extraversion (e); Agreeableness (a); Conscientiousness (c); Neuroticism (n); and Openness (o). These traits are typical personality constructs used in psychology and elsewhere (see Costa and McCrae, 1992; John and Srivastava, 1999). Each construct is measured by four self-report items (i.e., e1-e4, a1-a4, c1-c4, n1-n4, and o1-o4) on 5-point Likert-type scales, with responses anchored at 1 (strongly disagree) and 5 (strongly agree). The resulting repeating sequence for the items is: e1; a1; c1; n1; o1; e2; a2; c2; n2; o2; e3; a3; c3; n3; o3; e4; a4; c4; n4; o4 (for details see Donnellan et al., 2006 and http://ipip.ori.org/MiniIPIPKey.htm).

## Results

All models were estimated using Mplus version 8 (Muthén and Muthén, 1998–2017), with Mplus input, output, and data for all models available as a.zip file in online material [with annotated code in Online Appendix A (Supplementary Material)]. To examine model fit, we use typical ML fit statistics, including confirmatory fit index (CFI), Tucker-Lewis index (TLI), root mean squared error of approximation (RMSEA), and standardized root mean square residual (SRMR; see Hu and Bentler, 1998, 1999). We report Akaike's and the Bayesian information criterion (AIC and BIC; lower values are better), as typical for AR model selection (Akaike, 1969; Schwarz, 1978; Hannan and Quinn, 1979; Vrieze, 2012). For Bayesian analyses, we provide typical fit criteria including posterior-predictive probabilities (PPP; values around 0.5 are optimal and below 0.05 is problematic) and their associated χ^2^ 95% CIs (better when including zero, similar to traditional CFA model fit), as well as a deviance information criterion (DIC; lower values are better; Muthén and Asparouhov, 2012; Asparouhov et al., 2015). To facilitate contrasts with models that could only be estimated using a Bayesian approach, we also derived PPP and DIC values for models estimated using ML by separately subjecting these to model runs using a Bayesian estimator.

All models converged with some special considerations for two Bayesian models. For the Bayes AR-CFAs, we used a small-variance constraint on residuals to aid convergence—this does not impact results because residuals are freely estimated as latent variables. For the Bayes CFA with all possible cross-loadings (CL-CFA), we used small-variance priors of 0.01, and for the Bayes CFA with all residual covariances (RC-CFA), we took analysis steps recommended in Asparouhov et al. (2015; for details, see Online Appendix B in Supplementary Material).

Table 1 reports descriptive statistics^1^, Table 2 has fit statistics, Tables 3, 4 show factor correlations, factor loadings, and residual variances, and Table 5 shows AR terms from the AR-CFA. The presence of AR effects implies a simplex pattern of decreasing correlations away from the diagonal that could theoretically be observed in a correlation matrix. Given that all items have substantive meaning and that AR terms may vary in magnitude and differ in sign, however, this may not always be straightforward to observe. Nevertheless, in Table 1, where items are ordered by their factors (rather than in the order they were administered) an AR pattern would be indicated by higher correlations between e1 and a1, c1, n1, and o1, respectively, compared to e1 with a2, a3, and a4, c2, c3, and, c4 and so on, and by generally decreasing correlations in the item columns, some evidence of which can be observed in the e1 column and throughout the table.

**Table 1:** Descriptive statistics and correlations.

**Items**	***SD***	**Extraversion**				**Agreeableness**				**Conscientiousness**				**Neuroticism**				**Openness**			
		**e1**	**e2**	**e3**	**e4**	**a1**	**a2**	**a3**	**a4**	**c1**	**c2**	**c3**	**c4**	**n1**	**n2**	**n3**	**n4**	**o1**	**o2**	**o3**	**o4**
e1	1.13	1.00																			
e2	1.18	0.41	1.00																		
e3	1.22	0.50	0.40	1.00																	
e4	1.14	0.47	0.50	0.45	1.00																
a1	0.90	0.12	0.10	0.14	0.02	1.00															
a2	1.07	0.03	0.16	0.07	0.10	0.29	1.00														
a3	1.08	0.09	0.07	0.16	0.02	0.43	0.24	1.00													
a4	0.92	0.14	0.26	0.20	0.26	0.34	0.38	0.22	1.00												
c1	1.17	0.03	−0.01	0.07	−0.01	0.16	0.02	0.10	0.05	1.00											
c2	1.28	−0.03	0.02	−0.01	0.04	0.04	0.08	−0.01	0.08	0.32	1.00										
c3	1.03	−0.03	−0.02	0.02	−0.06	0.16	0.03	0.09	0.06	0.36	0.26	1.00									
c4	1.09	−0.02	0.05	0.02	0.09	0.08	0.08	−0.03	0.18	0.32	0.40	0.29	1.00								
n1	1.19	−0.01	−0.08	−0.09	−0.10	0.02	−0.05	0.09	−0.11	−0.04	−0.12	0.00	−0.23	1.00							
n2	1.10	−0.10	0.05	−0.15	−0.02	−0.10	0.00	0.00	−0.05	−0.03	−0.01	0.02	−0.10	0.31	1.00						
n3	1.19	−0.04	−0.04	−0.10	−0.11	0.05	−0.01	0.12	−0.08	0.00	−0.08	0.11	−0.19	0.48	0.35	1.00					
n4	1.12	−0.10	−0.04	−0.12	−0.05	−0.02	0.02	0.03	−0.01	−0.05	−0.02	−0.05	−0.06	0.25	0.24	0.21	1.00				
o1	1.08	0.15	0.08	0.15	0.07	0.22	0.03	0.15	0.10	0.01	−0.06	0.03	−0.07	0.09	−0.11	0.00	−0.02	1.00			
o2	1.04	0.01	0.07	0.06	0.12	0.12	0.17	0.08	0.21	−0.05	0.04	−0.04	0.03	−0.04	−0.04	−0.10	0.04	0.25	1.00		
o3	1.01	0.07	0.09	0.10	0.16	0.11	0.11	0.05	0.20	−0.02	0.06	−0.03	0.11	−0.14	−0.11	−0.20	−0.01	0.25	0.46	1.00	
o4	1.06	0.10	0.14	0.11	0.17	0.14	0.10	0.06	0.22	−0.02	0.03	−0.01	0.09	−0.06	−0.07	−0.09	0.03	0.53	0.29	0.32	1.00

**Table 2:** Model fit statistics for alternative model specifications.

	**IC-CFA**	**CL-CFA**	**RC-CFA**	**EFA**	**AR-CFA**	**AR-CFA w/priors**
AIC	488895.76	–	–	486317.49	485524.31	–
BIC	489389.69	487434.04	484247.73	487234.80	486258.15	486267.77
CFI	0.80	–	–	0.82	0.89	–
TLI	0.76	–	–	0.66	0.84	–
RMSEA	0.07	–	–	0.08	0.05	–
SRMR	0.053	–	–	0.03	0.04	–
Chi-square (df)	6052.87 (160)	–	–	5199.41 (100)	3279.08 (126)	–
PPP	<0.00	<0.00	0.27	<0.00	<0.00	<0.00
95% CI	6631.37–6741.87	4031.15–4145.55	−46.00 to 78.93	4018.33–4130.06	3248.95–3355.12	3259.44–3373.61
DIC	488895.96	486327.98	482324.70	486317.96	485515.23	485528.65
pD	70.04	125.59	215.62	130.08	101.26	101.79

*CL-CFA, CFA with cross-loadings; RC-CFA, CFA with residual covariances; AR-CFR, Auto-regressive CFA; CFI, comparative fit index; TLI, Tucker Lewis index; RMSEA, root mean square error of approximation; SRMR, standardized root mean square residual; PPP, posterior predictive probability; 95% CI, 95% confidence interval; DIC, deviance information criterion; pD, posterior mean deviance. The CFI, TLI, RMSEA, SRMS, and Chi-Square statistics are based on maximum likelihood estimation; whereas the PPP, 95% CI, DIC, and pD are based on Bayes estimation. The AR-CFA (maximum likelihood) is estimated with residual variances of observed variables set to 0; whereas the Bayes AR-CFA and the AR-CFA w/priors are estimated with residual variances of observed variables set to 0.01 in order to assist convergence—this specification does not impact results*.

**Table 3:** Standardized Factor loadings, residual variances, and factor correlations for alternative model specifications.

**Items**	**CFA**		**CL-CFA**		**RC-CFA**		**EFA**		**AR-CFA**		**AR-CFA w/priors**	
	**FL**	**RV**	**FL**	**RV**	**FL**	**RV**	**FL**	**RV**	**FL**	**RV**	**FL**	**RV**
e1	0.68	0.54	0.68	0.54	0.66	0.56	0.68	0.53	0.63	1.00	0.68	1.00
e2	0.64	0.59	0.66	0.58	0.63	0.60	0.65	0.58	0.62	0.99	0.70	0.97
e3	0.67	0.55	0.63	0.56	0.66	0.56	0.64	0.55	0.83	0.93	0.73	0.95
e4	0.71	0.50	0.75	0.44	0.66	0.56	0.74	0.43	0.70	0.85	0.67	0.97
a1	0.64	0.60	0.76	0.43	0.59	0.65	0.74	0.43	0.60	0.99	0.81	1.00
a2	0.51	0.74	0.37	0.83	0.46	0.79	0.32	0.84	0.51	0.99	0.50	0.94
a3	0.51	0.74	0.60	0.65	0.46	0.79	0.57	0.65	0.66	0.97	0.48	0.99
a4	0.60	0.64	0.35	0.71	0.62	0.61	0.31	0.71	0.66	0.85	0.61	0.86
c1	0.56	0.69	0.53	0.70	0.57	0.68	0.50	0.70	0.46	0.99	0.48	0.99
c2	0.58	0.67	0.60	0.65	0.55	0.70	0.59	0.65	0.46	0.98	0.50	0.98
c3	0.49	0.76	0.50	0.72	0.48	0.77	0.48	0.72	0.73	0.98	0.68	0.98
c4	0.63	0.60	0.65	0.53	0.60	0.65	0.65	0.52	0.75	0.64	0.72	0.81
n1	0.69	0.53	0.68	0.53	0.60	0.64	0.66	0.54	0.72	1.00	0.71	1.00
n2	0.49	0.76	0.52	0.75	0.46	0.79	0.53	0.72	0.61	0.95	0.59	0.97
n3	0.69	0.52	0.71	0.48	0.57	0.68	0.70	0.48	0.61	0.94	0.62	0.94
n4	0.35	0.87	0.35	0.88	0.37	0.86	0.36	0.86	0.38	1.00	0.38	1.00
o1	0.63	0.60	0.61	0.60	0.63	0.61	0.49	0.68	0.75	0.91	0.76	0.92
o2	0.50	0.75	0.51	0.74	0.48	0.77	0.59	0.65	0.41	0.99	0.40	0.99
o3	0.52	0.73	0.51	0.69	0.50	0.75	0.59	0.62	0.40	0.86	0.39	0.86
o4	0.71	0.50	0.74	0.48	0.68	0.53	0.62	0.60	0.70	0.99	0.69	0.99
**Factor Correlations**												
E ↔ A	0.31		0.24		0.39		0.21		0.27		0.24	
E ↔ C	0.03		0.05		0.04		0.04		0.01		0.01	
E ↔ N	−0.17		−0.18		−0.14		−0.15		−0.16		−0.15	
E ↔ O	0.22		0.27		0.27		0.23		0.23		0.24	
A ↔ C	0.23		0.13		0.25		0.10		0.15		0.19	
A ↔ N	−0.02		0.08		−0.07		0.07		−0.01		−0.04	
A ↔ O	0.38		0.25		0.42		0.19		0.32		0.33	
C ↔ N	−0.20		−0.21		−0.16		−0.21		−0.15		−0.18	
C ↔ O	0.02		0.05		0.02		0.05		0.01		0.00	
N ↔ O	−0.15		−0.20		−0.11		0.14		−0.13		−0.12	

*FL, factor loadings; RV, residual variances. EFA factor loadings and residuals are given after Geomin (oblique) rotation. Residual variances in the AR-CFA model are set to 0. Residual variances in AR-CFA w/priors are set to 0.01. RVs for both AR-CFA models refer to residuals of the respective auto-regressive factors specified in the model. Cross-loadings on other factors are not displayed due to space restrictions*.

**Table 4:** Average factor correlations for alternative model specifications.

**Factor correlations (in absolute value)**	**CFA**	**CL-CFA**	**RC-CFA**	**EFA**	**AR-CFA**	**AR-CFA prior**
E ↔ A	0.32	0.24	0.41	0.21	0.27	0.24
E ↔ C	0.03	0.05	0.04	0.04	0.01	0.01
E ↔ N	0.17	0.18	0.14	0.15	0.16	0.15
E ↔ O	0.22	0.28	0.28	0.23	0.23	0.24
A ↔ C	0.23	0.13	0.26	0.10	0.15	0.19
A ↔ N	0.02	0.08	0.07	0.07	0.01	0.04
A ↔ O	0.40	0.26	0.45	0.19	0.32	0.34
C ↔ N	0.20	0.21	0.16	0.21	0.15	0.18
C ↔ O	0.02	0.05	0.02	0.05	0.01	0.00
N ↔ O	0.15	0.20	0.11	0.14	0.13	0.12
Average factor correlations	0.18	0.17	0.19	0.14	0.15	0.15
Average factor correlations (in *r* metric)	**0.18**	**0.17**	**0.19**	**0.14**	**0.15**	**0.15**

*Average factor correlations in r metric represent Fisher transformation of the absolute value of the correlations from Table 3*.

**Table 5:** AR parameter estimates and interaction (Int) effects.

**AR Term**	**Effect**	***SE***	***t***	***p***	**Effect/Int**	**Int *SE***	**Int *t***	**Int *p***	**Item**
**Between-Construct Effects**									
									e1: Am the life of the party
e1 → a1°	0.06	0.012	5.063	<0.001					a1: Sympathize with others' feelings
a1 → c1°	0.137	0.02	6.97	<0.001					c1: Get chores done right away
c1 → n1°	0.035	0.012	3.036	0.002					n1: Have frequent mood swings
n1 → o1°	0.256	0.026	9.716	<0.001					o1: Have a vivid imagination
o1 → e2	−0.126	0.026	−4.789	<0.001	<0.13/0.017	0.033	0.518	0.605	e2: Don't talk a lot. R
e2 → a2	0.102	0.016	6.422	<0.001	0.103/0.008	0.018	0.421	0.674	a2: Am not interested in other people's problems. R
a2 → c2	0.09	0.016	5.567	<0.001	0.091/−0.009	0.022	−0.401	0.688	c2: Often forget to put things back in their proper place. R
c2 → n2	0.031	0.011	2.835	0.005					n2: Am relaxed most of the time. R
n2 → o2	−0.011	0.017	−0.637	0.524					o2: Am not interested in abstract ideas. R
o2 → e3	−0.021	0.023	−0.891	0.373	−0.015/−0.022	0.016	−1.417	0.157	e3: Talk to a lot of different people at parties
e3 → a3	0.11	0.052	2.112	0.035	0.113/0.034	0.067	0.51	0.61	a3: Feel others' emotions
a3 → c3	0.062	0.015	4.211	<0.001	0.063/0.028	0.02	1.406	0.16	c3: Like order
c3 → n3	0.302	0.024	12.466	<0.001					n3: Get upset easily
n3 → o3	−0.126	0.014	−9.175	<0.001					o3: Have difficulty understanding abstract ideas. R
o3 → e4	0.11	0.019	5.691	<0.001	0.103/0.003	0.017	0.16	0.873	e4: Keep in the background. R
e4 → a4	0.147	0.022	6.561	<0.001	0.149/0.019	0.022	0.852	0.394	a4: Am not really interested in others. R
a4 → c4	0.189	0.027	7.054	<0.001	0.181/−0.42	0.027	−1.535	0.125	c4: Make a mess of things. R
c4 → n4	0.058	0.023	2.554	0.011					n4: Seldom feel blue. R
n4 → o4	0.054	0.01	5.421	<0.001					o4: Do not have a good imagination. R
**Within-Construct Effects**									
									e1: Am the life of the party
e1 → e2	0.041	0.167	0.246	0.806	−0.017/−0.038	0.032	−1.209	0.227	e2: Don't talk a lot. R
e2 → e3	−0.198	0.068	−2.911	0.004	−0.213/−0.056	0.024	−2.325	0.02	e3: Talk to a lot of different people at parties
e3 → e4	−0.42	0.534	−0.787	0.431	−0.703/−0.082	0.042	−1.941	0.052	e4: Keep in the background. R
									a1: Sympathize with others' feelings
a1 → a2	−0.034	0.058	−0.582	0.561	−0.035/−0.009	0.028	−0.315	0.752	a2: Am not interested in other people's problems. R
a2 → a3	−0.122	0.02	−6.037	<0.001	−0.121/−0.047	0.018	−2.568	0.01	a3: Feel others' emotions
a3 → a4	−0.303	0.081	−3.757	<0.001	−0.296/0.001	0.022	0.051	0.959	a4: Am not really interested in others. R
									c1: Get chores done right away
c1 → c2	0.147	0.026	5.605	<0.001	0.143/0.045	0.019	2.366	0.018	c2: Often forget to put things back in their proper place. R
c2 → c3	−0.083	0.015	−5.623	<0.001	−0.086/−0.028	0.014	−2.017	0.044	c3: Like order
c3 → c4	−0.584	0.142	−4.103	<0.001	−0.682/−0.111	0.026	−4.27	<0.001	c4: Make a mess of things. R
									n1: Have frequent mood swings
n1 → n2	−0.242	0.091	−2.661	0.008					n2: Am relaxed most of the time. R
n2 → n3	−0.065	0.035	−1.874	0.061					n3: Get upset easily
n3 → n4	−0.032	0.035	−0.904	0.366					n4: Seldom feel blue. R
									o1: Have a vivid imagination
o1 → o2	−0.128	0.039	−3.246	0.001					o2: Am not interested in abstract ideas. R
o2 → o3	0.336	0.014	23.457	<0.001					o3: Have difficulty understanding abstract ideas. R
o3 → o4	0.057	0.02	2.894	0.004					o4: Do not have a good imagination. R

*Effects are raw regression weights, wherein e, extraversion; a, agreeableness; c, conscientiousness; n, neuroticism; o, openness; Int, a latent interaction effect among neuroticism and the residual AR predictor, °, an AR effect that should be ignored because it does not have an associated within-construct effect due to being an early item in the scale (e.g., in the e1a1 relationship there is no control for a past agreeableness effect, unlike the e2a2 effect for which there is an a1a2 effect). The Effect/Int column presents AR effects and their associated interactions from separate model runs for each residual treated as an outcome (models involving an AR effect for neuroticism's residual did not converge due to the fact that neuroticism is collinear with the outcome residual). The actual survey items associated with an AR effect are presented in the last column to aid the reader in understanding the associated AR effect*.

In what follows, we first compare model fit statistics and measurement characteristics for all models. Given the AR-CFA's favorable results, we then interpret AR effects and test hypotheses related to them, including their moderation by neuroticism and by using parameter constraints that help address concerns about overfitting. For chi-square difference tests of nested models, we used Satorra-Bentler chi-square correction factors (Satorra, 2000). For the interested reader we report chi-square differences for non-nested models, but note that in these cases *p*-values should not be understood as reflecting typical null hypothesis tests.

### Model Fit and Measurement Characteristics

We first estimated a typical IC-CFA as a baseline for model comparison. As expected, this model demonstrated problems with model fit: χ^2^ = 6052.87, *df* = 160, *p* < 0.001; CFI = 0.80; TLI = 0.76; RMSEA = 0.07; SRMR = 0.05; AIC = 488895.76; BIC = 489389.69; DIC = 488895.96; PPP <0.00. We then estimated an AR-CFA for comparison with the IC-CFA as well as other models. The *df* difference is 34, because with 20 items, there are 19 sequential “between-construct” AR terms, and with four items per factor, there are three AR terms per factor, for a total of 15 “within-construct” AR terms (recall that AR parameters are freely estimated—model output and annotated Mplus code can be found in Online Appendices). With the addition of the AR terms, model fit improved: χ^2^ = 3279.08, *df* = 126, *p* < 0.001; CFI = 0.89; TLI = 0.84; RMSEA = 0.05; SRMR = 0.04; AIC = 485524.31; BIC = 486258.15; DIC = 485515.23; PPP <0.001. The differences between the nested IC-CFA and the AR-CFA show improved fit in all indices (with positive Δ values indicating better AR-CFA fit, e.g., higher CFI or lower AIC): Δχ^2^ = 2363.90, Δ*df* = 34, *p* < 0.001; ΔCFI = 0.09; ΔTLI = 0.08; ΔRMSEA = 0.02; ΔSRMR = 0.01; ΔAIC = 3371.45; ΔBIC = 3131.54; ΔDIC = 3380.73; ΔPPP = 0. These results point to the benefits of a AR parameters for model fit, but other model comparisons are also useful for interpreting the AR-CFA.

For example, the AR-CFA can be compared to a non-nested alternative model with equivalent *df*, where all AR terms are reparameterized as autocorrelated residuals, which resulted in worse fit compared to the AR-CFA (with positive values in differences indicating better fit for the AR-CFA model): Δχ^2^ = 137.96, Δ*df* = 0, *p* < 0.001; ΔCFI = 0; ΔTLI = 0.01; ΔRMSEA = 0.01; ΔSRMR = 0; ΔAIC = 382.53; ΔBIC = 382.52; ΔDIC = 358.86; ΔPPP = 0. These results suggest that residuals may have indirect effects via AR terms that cannot be accounted for by residual correlations, partially supporting an AR-CFA wherein responses along past items affects future responses both directly and indirectly via AR paths.

Beyond this, the IC-CFA shows worse fit across most models and their indices (with positive Δ values indicating better fit for models other than the IC-CFA), including EFA^2^ (Δχ^2^ = 853.46, Δ*df* = 60, *p* < 0.001; ΔCFI = 0.02; ΔTLI = −0.1; ΔRMSEA = −0.01; ΔSRMR = 0.02; ΔAIC = 2578.27; ΔBIC = 2154.89; ΔDIC = 2578; ΔPPP = 0), Bayes CL-CFA (ΔBIC = 1955.65; ΔDIC = 2567.98; ΔPPP = 0), Bayes RC-CFA (ΔBIC = 5141.96; ΔDIC = 6571.26; ΔPPP = 0.27), as well as Bayes AR-CFA with small-variance priors of 0.01 and *M* = 0 on AR terms (ΔDIC = 3367.1; ΔPPP = 0). These results could be used to infer that indicators with common wordings or context impact model fit (e.g., CL-CFA), or the AR-CFA specification can capture serial dependence even when researchers do not *a priori* expect AR effects.

Yet, because some researchers are wary of models that include cross-loadings and residual specifications (e.g., Stromeyer et al., 2015), it is relevant to observe how the AR-CFA compares to these other models. Specifically, the AR-CFA shows mixed changes in fit across these specifications, with improved fit along most indices (RMSEA, CFI, TLI, and DIC) when compared to an EFA (with positive Δ values indicating better fit for the AR-CFA; Δχ^2^ = 1920.33, Δ*df* = 26, *p* < 0.001; ΔCFI = 0.07; ΔTLI = 0.18; ΔRMSEA = 0.03; ΔSRMR = −0.01; ΔAIC = 793.18; ΔBIC = 976.65; ΔDIC = 802.73; ΔPPP = 0), Bayes CL-CFA (ΔBIC = 1175.89; ΔDIC = 812.75; ΔPPP = 0), Bayes AR-CFA with small-variance priors and *M* = 0 on AR terms (ΔBIC = 9.62; ΔDIC = 13.42; ΔPPP = 0), but worse fit compared to Bayes RC-CFA (ΔBIC = −2010.42; ΔDIC = −3190.53; ΔPPP = −0.27). Indeed, the Bayes RC-CFA is superior to the AR-CFA in terms of BIC, DIC and the χ^2^ CI, showing good fit by including zero (95% CI for RC-CFA: −46 to 78.93). Further, AR-CFA Bayes model's 95% CI values are narrower and/or does not contain zero when compared to other Bayes CFAs (i.e., CL-CFA, RC-CFA, and AR-CFA with small-variance priors and *M* = 0).

Thus, although an AR-CFA does not represent an overall best fit across all models and fit indices (particularly compared to a RC-CFA), the AR-CFA does show improved fit across most indices when compared to models such as the IC-CFA and EFA, both of which are very common. Specifically, in relation to model fit, we infer that underlying AR effects among items may be a reasonable conclusion in the observed data, thereby lending some support for the proposition that items in a scale have an AR effect on subsequent items (similar to Krosnick, 1999). This result is important because, as we note above, the AR-CFA is a theoretically justified approach to modeling covariance among items in a scale, an issue that may deter some researchers from using an RC-CFA or other models.

To further interrogate and explore results with the AR-CFA, we now treat differences in standardized factor loading patterns and structural parameters in the form of latent factor correlations. When compared to an IC-CFA, the AR-CFA loadings demonstrate that several loadings increase above 0.70 due to the change in residual parameterization. As we describe in the next paragraph, one interpretation of this change is that the AR-CFA shows improved validity in a measurement-model sense (Bollen, 1989). For example, the loadings of e3, a3, a4, c3, c4, n1, n2, n4, and o1 in the IC-CFA changed from 0.67, 0.51, 0.60, 0.49, 0.63, 0.69, 0.49, 0.35, and 0.63 to consistently higher values in the AR-CFA of 0.83, 0.66, 0.66, 0.73, 0.75, 0.72, 0.61, 0.38, and 0.75, respectively (Table 3). Indeed, in the IC-CFA, all loadings are below 0.70 except for e4 and o4 items. Note that these increases are not a necessary outcome of moving from an IC-CFA to an AR-CFA; rather, the increases occur because modeling the AR effect reveals stronger relationships among items and their respective constructs.

Furthermore, compared to the IC-CFA, the AR-CFA latent factor correlations are smaller (Tables 3, 4), indicating better discriminant validity. It is possible that this is due to the unmodeled AR relationships in the IC-CFA inflating estimates of structural correlations among the latent variables. Also, the authors of the scale (Donnellan et al., 2006) deliberately selected items so that personality traits showed as high discriminant validity as possible while also balancing the reliability and content representativeness of the traits. Finally, when we look at factor variances in the AR-CFA except for factor C, we observe an increase in explained observed variance by each factor. This is important to note, because larger latent factor variances with an AR-CFA model suggest that a typical IC-CFA (that does not account for AR effects) might be demonstrating deflated factor variances in the structural model, making measurement scales appear less valid.

Regarding factor correlations, we also calculated average factor correlations in absolute value (Table 4). The results indicate that AR-CFA model has the lowest average (in an *r* metric) as 0.15 when compared to other CFA models: CFA = 0.18, CL-CFA = 0.17, and RC-CFA = 0.19. The lower average factor correlations with AR-CFA indicate better discriminant validity and can strengthen the effectiveness of diagnostic feedback (Marsh et al., 2010). The larger factor correlations can also signal bias that impacts structural relations.

In addition, when comparing the AR-CFA results to EFA, Bayes CL-CFA, Bayes RC-CFA, and Bayes AR-CFA with small variance priors and *M* = 0, the AR-CFA standardized loadings are larger for several items (Table 3; i.e., e3, a3, a4, c3, c4, n1, n2, n4, o1), similar size for some items (Table 3; e.g., e2, e4, a2, o4) and smaller for remaining items, indicating no obvious pattern of differences across the models. However, the AR-CFA does demonstrate improved discriminant validity with some smaller latent factor correlations when compared to CFA models. For example, compared to the IC-CFA, half of the latent factor correlations improved (i.e., decreased for E-A, E-C, A-C, A-O, and C-O), although three factor correlations remain approximately the same (i.e., E-N, E-O, and A-N). Thus, the average of factor correlations for AR-CFA models (AR-CFA and AR-CFA with prior values) are 0.15 (Table 4), which are smaller than CFA models (CFA = 0.18, CFA-CL = 0.17, RC-CFA = 0.19; see Table 4).

In sum, our results suggest that an AR-CFA shows improved fit when compared to other common models, including the IC-CFA and EFA. Also, standardized factor loadings increase and latent factor correlations decrease, indicating improved discriminant validity. Finally, when compared to alternative models using a Bayes estimator, mixed results are found using DIC and the χ^2^ CIs, with some models showing improved fit, perhaps at the expense of theoretical justification (including the Bayesian CL-CFA and RC-CFA). In sum, our results indicate that when researchers use CFA to evaluate measurement model fit (Bollen, 1989), an AR-CFA may not provide optimal fit when compared to all available models, but given its theoretical justification and the improved fit it shows beyond typical IC-CFA and EFA models, we suggest that the AR-CFA is a potentially useful option for researchers.

### Interpreting AR Effects and Hypothesis Tests

We now offer a substantive interpretation of the AR parameters in Table 5 and then proceed to test additional hypotheses about them using averages of AR parameters and model constraints. To interpret AR parameters, it is important to point out that when there are multiple AR effects impacting an outcome variable, such as the between- and within-construct effects in our model, interpreting effects is usually only done when an outcome variable is impacted by all AR effects of interest. For example, the first a1 item has a between-construct effect of the previous e1 item (i.e., e1 → a1). However, each subsequent a2, a3, and a4 item has a between-construct effect of the previous e2, e3, and e4 item (i.e., e2 → a2, e3 → a3, and e4 → a4), *and* a within-construct effect of the previous a1, a2, and a3 item (i.e., a1 → a2, a2 → a3, and a3 → a4). Thus, only the e2 → a2, e3 → a3, and e4 → a4 effects are equivalent, and therefore we focus on such effects for hypothesis testing and model constraints (see the ° symbol in Table 5).

As Table 5 shows, although small in magnitude, most AR effects are statistically significant—this is unsurprising given the large sample size. More important is the fact that the small magnitudes indicate that the AR effects will fade relatively quickly across items (e.g., e2 → a2 = 0.102 and a2 → c2 = 0.09, so the e2 → a2 → c2 effect = 0.0092). This mitigates concerns about AR effects accounting for too much residual covariation because, as we noted previously, the multiplicative nature of the AR structure reflects rather temporary effects that decay as new items are answered over time (i.e., indirect AR effects fade rather quickly).

Examining the between-construct AR effects, most of them appear reasonable from a substantive perspective. Consider our previous example of the e2 → a2 relationship among the “Don't talk a lot” and “Am not interested in other people's problems” items. This relationship is positive (AR effect = 0.102, *SE* = 0.016, *t* = 6.422, *p* < 0.001), which implies that responses to the extraversion item that deviate from one's true score along extraversion (i.e., higher scores along the e2 residual) persist, leading to slightly higher scores on the agreeableness item that cannot be accounted for by the true score along agreeableness (i.e., higher scores along the a2 residual). To understand this, it is reasonable to presume that respondents may reflect on a time that they did not talk a lot in an interpersonal encounter, which they may then infer implies that they are not interested in others' problems and/or may more easily lead to a memory of being disinterested in others' problems. The affect generated by thinking about the experience combined with any similarly activated memory could create the small e2 → a2 effect observed here, such that endorsing the previous item could lead to endorsing the latter in a manner that is not accounted for by latent standings along the factors themselves.

Turning to the within-construct effects, again most (although not all) of the effects were statistically significant. More interesting was the fact that many of these effects were negative. For example, the e2 → e3 effect of the “Don't talk a lot” item and the subsequent extraversion items “Talk to a lot of different people at parties” was negative and statistically significant (AR effect = −0.198, *SE* = 0.068, *t* = −2.911, *p* = 0.004). This runs counter to the way we hypothesized within-construct effects—we presumed that cognitive and affective priming would create positive AR effects—but the pattern of effects in Table 5 does shed light on a potentially interesting phenomenon.

In most cases, the mini-IPIP scale presents items for all five factors such that the first set of items is normally coded and then the subsequent set is reverse-coded, and so forth. With this pattern, Table 5 shows that when items are reversed the AR effect of normal-reverse (or reverse-normal) coded item pairs is often negative (e.g., the e2 → e3 effect). Instead of such residual covariance indicating a “method” factor associated with negatively worded items (as is sometimes hypothesized), it is possible that the reversal creates what Tourangeau and Rasinski (1988) refer to as a “backfire” effect, wherein responses to a past item somehow disrupt future item responses. For example, a negative AR effect may arise as people grapple with the fact that the previous item coupled their thoughts and feelings to the opposite end of the scale, which may pull their future responses closer to that end of the scale. In turn, when reverse-coded items are unreversed for data analysis, this would induce a negative AR effect among the items—we show how to test this momentarily.

Next, consider our hypothesis about moderation by neuroticism, which we proposed would make AR effects larger. This hypothesis would be supported by a main AR effect and an interaction effect that are the same sign (i.e., a positive AR effect would be more positive if neuroticism were higher, and a negative AR effect would be more negative if neuroticism were higher). Although only five of the AR effects in Table 5 were moderated by neuroticism with *p* < 0.05, all of these showed a main AR effect and an interaction effect that were the same sign. Also, across all interactions, 14 of the 18 had a main effect and an interaction effect that were the same sign, which tentatively supports our moderation hypothesis.

#### Average AR Effects and Model Constraints

Next, it is possible to examine the AR effects in an AR-CFA in two additional ways. First, overall averages of AR effects can be computed to test omnibus hypotheses about similar AR effects in a model (e.g., the e1 → e2, e2 → e3, and e3 → e4 effects; see Online Appendix A in Supplementary Material). These effects are computed as follows for the between-construct effects: e → a = 0.12, *SE* = 0.012, *t* = 10.422, *p* < 0.001; a → c = 0.114, *SE* = 0.009, *t* = 13.236, *p* < 0.001; c → n = 0.13, *SE* = 0.011, *t* = 12.008, *p* < 0.001; n → o = −0.027, *SE* = 0.008, *t* = −3.535, *p* < 0.001; o → e = −0.012, *SE* = 0.13, *t* = −0.937, *p* = 0.349. For the within-construct effects we find: e → e = −0.192, *SE* = 0.101, *t* = −1.901, *p* = 0.057; aa = −0.153, *SE* = 0.014, *t* = −10.988, *p* < 0.001; cc = −0.173, *SE* = 0.04, *t* = −4.341, *p* < 0.001; n → n = −0.113, *SE* = 0.027, *t* = −4.215, *p* < 0.001; o → o = 0.088, *SE* = 0.013, *t* = 6.583, *p* < 0.001. Taken together, these appear to show, on average, AR effects that are predictably small but systematic. However, computing an average of AR effects does not imply that similar AR effects are actually the same.

To test for equivalence in similar AR effects, we conducted a series of nested model comparisons by constraining common AR effects to equality. The first set of constraints took the average AR effects as a starting point, so that instead of computing averages each set of effects were constrained to equality, resulting in a single effect for each e → a, a → c, c → n, n → o, o → e, e → e, a → a, c → c, n → n, and o → o effect (see Online Appendix A in Supplementary Material). This impacted model fit compared to the unrestricted AR-CFA, indicating that the AR parameters for each item pairing were different (positive values indicate better fit for the unconstrained vs. constrained model): Δχ^2^ = 914.97, Δ*df* = 20, *p* < 0.01; ΔCFI = 0.03; ΔTLI = 0.02; ΔRMSEA = 0.01; ΔSRMR = 0.02; ΔAIC = 1275.16; ΔBIC = 1134.03; ΔDIC = 1362.76; ΔPPP = 0. This indicates that similar AR parameters statistically differ from one another, implying that the content of unique item-item pairs drives AR effects more than the latent constructs with which items are associated. This is sensible to the extent that the latent personality factors will account for construct-specific (co)variance, with item-specific (co)variance remaining.

Although we did not hypothesize equality in similar AR effects, and therefore this did not come as a surprise, the rather sizable reduction in model fit led us to consider how the reverse-scoring of scale items might impact AR terms. As Table 5 shows, there are multiple ways that items can be connected via AR paths: normal → normal scoring; reverse → reverse scoring; normal → reverse scoring; and reverse → normal scoring. With such relationships, there are other ways to constrain AR terms that are sensitive to item scoring.

To show this, we constrained AR effects to equality only when the pattern of item scorings was similar (i.e., normal → normal and reverse → reverse scored item AR effects were constrained to equality; and normal → reverse and reverse → normal scored item AR effects were constrained to equality; see Online Appendix A in Supplementary Material). Compared to the previous differences in model fit above, this model compared more favorably to the original AR-CFA (positive values indicate better fit for the unconstrained vs. constrained model): Δχ^2^ = 588.82, Δ*df* = 17, *p* < 0.01; ΔCFI = 0.02; ΔTLI = 0.01; ΔRMSEA = 0.01; ΔSRMR = 0.01; ΔAIC = 896.66; ΔBIC = 776.71; ΔDIC = 902.58; ΔPPP = 0. As this shows, model fit improves somewhat by being more sensitive to reverse scoring, which points to potential “backfire” effects wherein AR terms change as a function of item scoring (see Tourangeau and Rasinski, 1988).

Specifically, the effects for similarly scored items are (i.e., normalnormal scored item AR effects): e → a = 0.132, *SE* = 0.009, *t* = 15.484, *p* < 0.001; a → c = 0.099, *SE* = 0.009, *t* = 11.49, *p* < 0.001; c → n = 0.103, *SE* = 0.009, *t* = 12.136, *p* < 0.001; n → o = 0.034, *SE* = 0.008, *t* = 4.056, *p* < 0.001; o → e = 0.117, *SE* = 0.012, *t* = 9.498, *p* < 0.001; o → o = 0.217, *SE* = 0.012, *t* = 17.661, *p* < 0.001. All of these effects are positive, which is interesting in light of the following negative effects for items that are differently scored (i.e., normal → reverse and reverse → normal scored item AR effects): no = −0.156, *SE* = 0.014, *t* = −11.155, *p* < 0.001; o → e = −0.044, *SE* = 0.011, *t* = −3.81, *p* < 0.001; e → e = −0.13, *SE* = 0.011, *t* = −11.63, *p* < 0.001; aa = −0.118, *SE* = 0.015, *t* = −8.064, *p* < 0.001; c → c = −0.075, *SE* = 0.013, *t* = −5.607, *p* < 0.001; n → n = −0.102, *SE* = 0.015, *t* = −6.893, *p* < 0.001; o → o = −0.335, *SE* = 0.049, *t* = −6.801, *p* < 0.001. Although this pattern may be due to the fact that many of the similarly scored items are between-construct AR effects whereas the differently-scored items are within-construct AR effects, it does appear that the scoring of items may systematically impact item AR effects.

### Monte Carlo Simulations

A simulation study with AR-CFA and IC-CFA models was conducted with varying sample sizes of 250, 500, and 1,000 as well as AR effects of 0, 0.1, 0.2, and 0.3 (when an AR effect = 0 in the population model this implies an IC-CFA, i.e., no AR effects are present). The factor loadings, residual variances and other population parameters were set by relying on typical standards of standardized loadings of 0.8 and factor correlations of 0.2 (Muthén and Muthén, 2002; Hallquist and Wiley, 2018; Ondé and Alvarado, 2018). In brief, our simulations show that the AR-CFA does not show parameter bias or meaningful differences in fit even if data are generated from an IC-CFA, so even if strict IC-CFA assumptions are met the AR-CFA may be useful. However, the IC-CFA over-estimates factor loadings and factor covariances as AR parameters increase in the population, which is consistent with our real-data example wherein the AR-CFA shows better discriminant validity. Next, the AR-CFA has convergence problems with samples of 250, particularly with small AR parameters, suggesting samples of at least 400–500 to estimate the AR-CFA (or perhaps using a Bayes estimator). Finally, the IC-CFA shows poorer model fit as AR parameter values get larger, with levels of IC-CFA misfit often found in practice, suggesting that unmodeled AR effects may be the cause of some observed misfit in the literature when using the IC-CFA. As expected, because a population model is not knowable in practice, this must remain conjecture. The reader can evaluate our simulation results more completely in Online Appendix D and our online Excel file that tabulates all simulation results.

## Discussion

Drawing on existing theory, we demonstrate the importance of sequential order effects in survey research. Specifically, our work is motivated by the possibility that typical CFA models (e.g., IC-CFA) should include both latent variable and AR effects, the latter of which allow past survey items to affect later item responses in ways that are unrelated to the latent factors of interest in a CFA. This sequential response process and its effects have been investigated by researchers (Schwarz, 1999, 2011) and hybrid longitudinal approaches to CFA-type models have been implied in literature on panel data analyses (see Bollen and Curran, 2004), but the AR-CFA approach we describe here is a novel way to model such effects.

By grounding AR effects in literature on item context effects, we theorize two key mechanisms that produce AR processes in a survey setting: memory activation and affective priming. Our conceptualization of these mechanisms and the potential moderating effects of stable traits such as neuroticism offer a sound theoretical rationale for expecting AR effects in survey data. By modeling AR effects among item residuals, our method permits operationalizing various kind of AR effects, as well as evaluating such effects by computing their averages or through model constraints—as we demonstrate here. In sum, the AR-CFA model offers improved fit and a more theoretically rigorous approach to model specification when compared to the IC-CFA, while also offering ways to balance the difference between highly a constrained IC-CFA vs. other less-constrained approaches such as an EFA.

Our substantive findings help to justify the AR-CFA by showing improved discriminant validity in the form of larger factor loadings and latent factor variances as well as smaller latent factor covariances and lower average factor correlations among personality traits. This is done while accounting for what could otherwise be problematic item-level covariances in form of lagged AR effects. Although many alternative measurement models are possible and our goal here is not to criticize these approaches, our comparisons against competing models (e.g., Bayes CL-CFA, Bayes RC-CFA), in conjunction with the theoretical justification for an AR-CFA give us some confidence in recommending it as a viable alternative to other approaches.

### Practical Implications

When considering the use of the AR-CFA by researchers or practitioners, two issues arise. The first relates to how scales should be scored if, at the item level, it is reasonable to expect relationships that deviate from an IC-CFA in ways that could bias, for example, scale means. On this point, it is important to point out that, like the RC-CFA, the AR-CFA specifies item-level relationships in a way that can be conceptually and mathematically separated from the latent factors that are typically of interest in a CFA. In turn, it becomes possible to use scoring methods that rely only on the latent factors themselves. These would include specifying an AR-CFA and then computing factor scores for latent factors of interest (Grice, 2001), but still better would be computing plausible values that account for uncertainty in latent standings along the factors (see Online Appendix A in Supplementary Material). This approach has recently been popularized in educational testing contexts (see von Davier et al., 2009) and can be implemented using a Bayesian estimator in Mplus.

The next issue to consider is the increasingly popular method of randomizing items when surveys are administered online (see Schell and Oswald, 2013). Using a similar logic to within-subjects experimental designs (see Shadish et al., 2002), the purpose of randomizing item order is to eliminate the kinds of AR effects we treat here. Although item randomization is a promising approach for dealing with context effects, given our treatment of AR effects here, randomization does seem to come with some unacknowledged assumptions. To explain, consider the following three cases: Case (1) there are no AR effects (i.e., no context effects); Case (2) AR effects exist they will cancel out to exactly zero due to item randomization; and Case (3) AR effects exist but they will not cancel out to zero due to item randomization.

Obviously, in Case 1 item randomization is not needed (Schell and Oswald, 2013), but it would also cause no harm in the long run, just as our AR-CFA would cause no harm because AR terms would converge to zero with well-functioning scales. More importantly, Case 2 would seem to be the basis for item randomization, because item randomization will only provide benefits when AR effects can be expected to exist yet average to zero in the long run across all possible combinations of item orderings. By averaging to zero in the long run, item randomization should produce unbiased and consistent CFA estimates—although it may reduce statistical efficiency because of the error (co)variance caused by person-specific AR effects. However, in this Case 2, our AR-CFA would also be justified without item randomization because the AR effects would be appropriately modeled, and efficiency in CFA estimates would be appropriately reduced because of the estimation of AR terms. Therefore, Case 2, for item randomization is designed, should provide a kind of indifference among randomization and an AR-CFA, because both can account for AR effects and appropriately adjust estimates of uncertainty in CFA parameter estimates.

However, consider Case 3, which to our knowledge has not been adequately treated. For this, consider a classic context effect (as suggested by Schwarz and Clore, 1983): Item 1 induces affect and Item 2 relies on affect for item responding, but not the reverse. Here, if the order of the items is randomized across participants the AR effect will not be eliminated, because a positive Item 1 → Item 2 AR effect and a zero Item 2 → Item 1 AR effect will average to an overall positive residual correlation due to the Item 1 → Item 2 effect (this effect is halved when half the participants receive each of the two orderings in equal numbers).

In more typical cases, depending on the items in a scale, it seems reasonable to propose that Case 3 might actually be more common than Case 2. Indeed, what if Case 3 is the rule rather than the exception? If Case 3 is common, randomization will cause bias, inconsistency, and inefficiency in CFA estimate due to non-zero average AR effects that might appear as true-score correlations. In this case, conveniently, our AR-CFA method used on items delivered without randomization can capture any AR terms of interest, and therefore it should eliminate any bias and inconsistency caused by randomization.

In sum, across all three of these Cases, the AR-CFA should perform as well or better than item randomization and, in Case 3, the AR-CFA is the only way to ensure unbiased and consistent CFA estimates because randomization fails to solve the problem of non-zero average AR terms. Certainly, as with all modeling efforts it is not possible to know which Case exists a priori, it is possible to theorize and test AR effects in an AR-CFA.

### Limitations and Future Research Directions

Our research challenges current procedures for specifying measurement models in SEM, serving to advance typical CFA methods based on theory about AR effects in survey responses. Our comparison models included IC-CFA and EFA as common measurement models, as well as Bayesian CFA models with residual covariances and small-variance priors. Given that Bayesian RC-CFA and potentially other similar models may show a better fit to the data, we cannot unconditionally recommend the AR-CFA for those seeking to optimize observed fit to a dataset. Furthermore, although we have emphasized the theoretical justification for the AR component in our model, it is notable that CL-CFA and RC-CFA are also theoretically justified (see Muthén and Asparouhov, 2012; Asparouhov et al., 2015)—although not all researchers appreciate this justification (e.g., Stromeyer et al., 2015).

Our study also attempts to join two streams of findings from existing literature: AR effects specified with panel data and IC-CFAs used to model connections between observed survey responses and underlying latent constructs. On this point, because of the necessarily fixed sequential nature of AR effects across items and survey respondents (i.e., all survey respondents have their data subjected to the same model structure), the AR-CFA we propose can only account for temporal AR effects when items are ordered in a fixed and shared way across items and respondents. This, the AR-CFA is useful for the many surveys that use a fixed ordering of questions, mailed survey databases, national surveys such as the Household Income and Labor dynamics in Australia (HILDA) dataset, or research that relies on historical datasets that were collected with fixed item orderings via questionnaires.

Future work can explore how adding item- and person-varying AR parameters may allow relaxing this constraint to treat the case of items being randomized across participants. This would be possible by specifying person-specific AR matrices using special parameter constraint methods (e.g., Mplus's “constraint” option). Certainly, in the case of such randomization, it is interesting to consider how this is an attempt to overcome the kind of order effects that our AR terms capture, but such randomization only does this by introducing unmodeled order effects that vary across participants in the form of unsystematic (and unmodeled) residual covariance. Instead of this, the AR-CFA may be a viable alternative to addressing the concerns that random item ordering across respondents is meant to achieve—a topic for future research by modeling specifying person-specific AR terms. Furthermore, it is worth noting that even though randomized-item surveys may “average out” AR effects at the level of a *population*, with a finite sample size this is not possible, and therefore even fully randomized item orderings should produce some bias, and in all cases will reduce efficiency and therefore lead to inflated standard errors. In addition to addressing this problem, future research may consider how item response times and other time-varying covariates can be incorporated into measurement models to examine the residual covariance induced by item orderings (extending related work by Klein Entink et al., 2009). One approach for testing such order effects is shown in Online Appendix C (Supplementary Material), which may serve as a potentially interesting way to examine how AR effects systematically change across items.

Furthermore, our AR-CFA in its current form offers only a single AR term for each type of item pairing, effectively implying a kind of single-order AR process linking indicator-item residuals. However, more complex, higher-order AR or moving-average (MA) processes allowing for more complex residual covariance structures could be implemented (for general insight, see Hamaker, 2005; Hamaker et al., 2005). Given the introductory nature of our paper, the topic of higher-order AR structures and MA structures can be the subject of future work, where more complex ARMA processes can be tested against simpler ones to see whether the incremental gain in model fit justifies their added complexity.

In conclusion, since the origination of EFA for modeling survey responses, there have been a vast array of methodological developments, culminating in confirmatory techniques that, today, take a wide variety of forms—most notably a highly restricted IC-CFA. Our AR-CFA is meant to extend and complement these existing approaches for those who seek to add a temporal element into their measurement models while improving model fit. We hope that the AR-CFA is useful for this purpose, and we look forward to future work on how it can be further developed and applied across a wide cross-section of settings, samples, and purposes.

## Ethics Statement

This study was exempt from ethics oversight, as per the University of Melbourne human subjects protocol, because the data are anonymized and exist in the public domain.

## Author Contributions

OO and MZ contributed equally to this project to formulate the idea, procure data, analyze it, and draft early versions of the manuscript. AB drafted the theory section and otherwise he, along with MT, MD, and FO, contributed equally to manuscript drafts and the general ideas associated with the project.

### Conflict of Interest

The authors declare that the research was conducted in the absence of any commercial or financial relationships that could be construed as a potential conflict of interest.

## Footnotes

^1^Regarding missing values, minimum covariance coverage value is 0.1. ML and Bayes estimation procedures are full-information methods that assume missing at random processes. Therefore, we did not encounter any isses regarding missing values as model estimates are based only on the observed data.

^2^EFA and ESEM have the same model structure because there is a fully saturated covariance structural model in both cases (as we specify for all models).
