# Supplementary material for: Modeling Measurement as a Sequential Process: Autoregressive Confirmatory Factor Analysis (AR-CFA)
Source: Front Psychol. 2019 Sep 20;10:2108. doi: 10.3389/fpsyg.2019.02108 (PMC6763968; doi:10.3389/fpsyg.2019.02108)
Supplement: Supplementary file 1 [file Table_1.docx]

Online Appendix A

Mplus Code for All Models

Here we offer Mplus code for all models, with model names as in our text. Comments follow exclamation points, with annotations to explain model input. Parameter labels are created using parentheses, which can be used for model constraints or model prior specifications (the latter for use with a Bayes estimator). We first provide general input statements that should be placed at the top of any Mplus input file. Following this, we offer model-specific input that should be added to the general input statements in any Mplus input file.

**GENERAL INPUT STATEMENTS**

! The model input here should be placed at the top of any Mplus input file

DATA: File is music_big5_anon (For Mplus)_ipip_and_rses_centered.csv;

VARIABLE: NAMES ARE

! The full list of variable names is reduced here to save space, but the full list is available in

! any of our online appendices containing Mplus input or output and can be substituted here

mipip1-mipip20; ! mini IPIP items 1-20 and scale means

Usevar = mipip1-mipip20; MISSING are all (-999);

OUTPUT: stdyx; ! Requests standardized output

**IC-CFA**

ANALYSIS: estimator=mlr; Processors=8;

MODEL:

E BY mipip1 mipip6 mipip11 mipip16; ! Defines latent extraversion variable ‘E’

A BY mipip2 mipip7 mipip12 mipip17; ! Defines latent agreeableness variable ‘A’

C BY mipip3 mipip8 mipip13 mipip18; ! Defines latent conscientiousness variable ‘C’

N BY mipip4 mipip9 mipip14 mipip19; ! Defines latent neuroticism variable ‘N’

O BY mipip5 mipip10 mipip15 mipip20; ! Defines latent openness variable ‘O’

**EFA**

ANALYSIS: estimator=mlr; Processors=8;

type=EFA 5 5; ! Indicates 5 factors are to be extracted with oblique rotation

**AR-CFA (MAXIMUM LIKELIHOOD)**

**!To specify an AR-CFA model in Mplus,**

**!Researchers first should start with an IC_CFA structure.**

**!Create new latent variables to capture each item’s residual variance.**

**! Then create an AR structure among these latent residuals (Formula 3 & 4)**

**!AR structure is created between and within constructs.**

**!When analysis is run, all factor loadings, latent factor variances and covariances, and !AR terms are freely estimated.**

ANALYSIS: estimator=mlr; Processors=8;

MODEL:

E BY mipip1 mipip6 mipip11 mipip16; ! Typical factor structure as before

A BY mipip2 mipip7 mipip12 mipip17;

C BY mipip3 mipip8 mipip13 mipip18;

N BY mipip4 mipip9 mipip14 mipip19;

O BY mipip5 mipip10 mipip15 mipip20;

! The following specification creates latent variables to capture item residuals

rmipip1 by mipip1@1; ! The latent residual is now rmipip with a ‘factor loading’ of 1

mipip1@0; ! Item residual fixed to zero

rmipip2 by mipip2@1; mipip2@0; rmipip3 by mipip3@1; mipip3@0; rmipip4 by mipip4@1; mipip4@0; rmipip5 by mipip5@1; mipip5@0; rmipip6 by mipip6@1; mipip6@0; rmipip7 by mipip7@1; mipip7@0;

rmipip8 by mipip8@1; mipip8@0; rmipip9 by mipip9@1; mipip9@0; rmipip10 by mipip10@1; mipip10@0; rmipip11 by mipip11@1; mipip11@0; rmipip12 by mipip12@1; mipip12@0; rmipip13 by mipip13@1; mipip13@0; rmipip14 by mipip14@1; mipip14@0;

rmipip15 by mipip15@1; mipip15@0; rmipip16 by mipip16@1; mipip16@0; rmipip17 by mipip17@1; mipip17@0; rmipip18 by mipip18@1; mipip18@0; rmipip19 by mipip19@1; mipip19@0; rmipip20 by mipip20@1; mipip20@0;

E A C N O with rmipip1; ! Fix correlation of factors and first residual to 0, else it’s estimated

! Lagged ‘between-construct’ AR terms representing context effects are as follows:

rmipip2-rmipip20 on rmipip1-rmipip19; ! Regresses each residual on previous residual

! E terms : Within-construct (factor) AR terms for E items

rmipip6 on rmipip1; rmipip11 on rmipip6; rmipip16 on rmipip11;

! A terms : Within-construct (factor) AR terms for A items

rmipip7 on rmipip2; rmipip12 on rmipip7; rmipip17 on rmipip2;

! C terms : Within-construct (factor) AR terms for C items

rmipip8 on rmipip3; rmipip13 on rmipip8; rmipip18 on rmipip13;

! N terms: Within-construct (factor) AR terms for N items

rmipip9 on rmipip4; rmipip14 on rmipip9; rmipip19 on rmipip14;

! O terms: Within-construct (factor) AR terms for O items

rmipip10 on rmipip5; rmipip15 on rmipip10; rmipip20 on rmipip15;

**CFA W/ CORRELATED RESIDUALS THAT MIRROR AR-CFA PATHS**

! For this model, everything is the same as the IC-CFA and AR-CFA, except the residuals

! now have the following under the MODEL command:

E A C N O rmipip1-rmipip20 with rmipip1-rmipip20@0; ! Residuals are unrelated to factors

! Lagged ‘between-construct’ correlated residuals

rmipip2-rmipip20 pwith rmipip1-rmipip19;

! Lagged ‘within-construct’ correlated residuals

! E terms

rmipip6 with rmipip1; rmipip11 with rmipip6; rmipip16 with rmipip11;

! A terms

rmipip7 with rmipip2; rmipip12 with rmipip7; rmipip17 with rmipip12;

! C terms

rmipip8 with rmipip3; rmipip13 with rmipip8; rmipip18 with rmipip13;

! N terms

rmipip9 with rmipip4; rmipip14 with rmipip9; rmipip19 with rmipip14;

! O terms

rmipip10 with rmipip5; rmipip15 with rmipip10; rmipip20 with rmipip15;

**AR-CFA W/ LATENT INTERACTIONS**

! For this model, everything is equivalent to the previous AR-CFA, except a separate model

! is run for each outcome in an AR equation, with the following changes to ANALYSIS

ANALYSIS: estimator=mlr;

Processors=2; type=random; algorithm=integration; integration=10;

! This sets numerical integration for the variables involved in the latent interaction. Then, the

! latent interactions are created and used as predictors in MODEL statement, such as:

MODEL:

! Include normal AR-CFA specification here, but for the outcome rmipip6 set the following:

int1 | n xwith rmipip5; ! Defines latent interaction among neuroticim and residual for mipip5

int2 | n xwith rmipip1; ! Defines latent interaction among neuroticim and residual for mipip1

rmipip6 on rmipip5 int1 rmipip1 int2; ! Regression equation for ripip6

! Then save results and rerun the model for rmipip7 (and its predictors) and so forth

**AR-CFA W/ PRIORS**

! All parameters will be equivalent to an AR-CFA, except priors will be put on AR terms

! The following estimation approach reduces the number of reported iterations to 10,000

! while actually allowing for 2,000,000 by thinning to select only every 200th iteration

ANALYSIS: estimator=Bayes; fbiterations=10000; thin=200; Processors=2; chains=2;

MODEL:

E BY mipip1 mipip6 mipip11 mipip16; ! Typical factor structure

A BY mipip2 mipip7 mipip12 mipip17;

C BY mipip3 mipip8 mipip13 mipip18;

N BY mipip4 mipip9 mipip14 mipip19;

O BY mipip5 mipip10 mipip15 mipip20;

! Creates latent variables for residuals

rmipip1 by mipip1@1; rmipip2 by mipip2@1; rmipip3 by mipip3@1; rmipip4 by mipip4@1; rmipip5 by mipip5@1; rmipip6 by mipip6@1; rmipip7 by mipip7@1; rmipip8 by mipip8@1; rmipip9 by mipip9@1; rmipip10 by mipip10@1; rmipip11 by mipip11@1; rmipip12 by mipip12@1; rmipip13 by mipip13@1; rmipip14 by mipip14@1; rmipip15 by mipip15@1; rmipip16 by mipip16@1; rmipip17 by mipip17@1; rmipip18 by mipip18@1; rmipip19 by mipip19@1; rmipip20 by mipip20@1;

E A C N O with rmipip1@0;

mipip1-mipip20@.01; ! Fixed small-variance to aid in convergence/MCMC chain mixing

! AR terms with parameter labels in parentheses to be used under MODEL PRIORS

rmipip2 on rmipip11 (a1); rmipip3 on rmipip2 (a2); rmipip4 on rmipip3 (a3); rmipip5 on rmipip4 (a4); rmipip6 on rmipip5 (a5); rmipip7 on rmipip6 (a6); rmipip8 on rmipip7 (a7); rmipip9 on rmipip8 (a8); rmipip10 on rmipip9 (a9); rmipip11 on rmipip10 (a10); rmipip12 on rmipip11 (a11); rmipip13 on rmipip12 (a12); rmipip14 on rmipip13 (a13); rmipip15 on rmipip14 (a14); rmipip16 on rmipip15 (a15); rmipip17 on rmipip16 (a16); rmipip18 on rmipip17 (a17); rmipip19 on rmipip18 (a18); rmipip20 on rmipip19 (a19);

! E terms

rmipip6 on rmipip1 (b1); rmipip11 on rmipip6 (b2); rmipip16 on rmipip11 (b3);

! A terms

rmipip7 on rmipip2 (b4); rmipip12 on rmipip7 (b5); rmipip17 on rmipip2 (b6);

! C terms

rmipip8 on rmipip3 (b7); rmipip13 on rmipip8 (b8); rmipip18 on rmipip13 (b9);

! N terms

rmipip9 on rmipip4 (b10); rmipip14 on rmipip9 (b11); rmipip19 on rmipip14 (b12);

! O terms

rmipip10 on rmipip5 (b13); rmipip15 on rmipip10 (b14); rmipip20 on rmipip15 (b15);

MODEL PRIORS: a1-b15~N(0,.01); ! Set’s priors to normal with M=0 and VAR=.01

OUTPUT: STDYX tech8; ! Tech8 requests iteration history for Bayes analysis

**CL-CFA**

ANALYSIS: estimator=Bayes; fbiterations=10000; thin=10; Processors=2; Chains=2;

MODEL:

! The following model sets up all possible cross-loadings with parameter labels for priors

E BY mipip1

mipip6

mipip11

mipip16

mipip2 (a1)

mipip7 (a2)

mipip12 (a3)

mipip17 (a4)

mipip3 (a5)

mipip8 (a6)

mipip13 (a7)

mipip18 (a8)

mipip4 (a9)

mipip9 (a10)

mipip14 (a11)

mipip19 (a12)

mipip5 (a13)

mipip10 (a14)

mipip15 (a15)

mipip20 (a16);

A BY mipip1* (b1) ! Frees first factor loading, typically fixed by default

mipip6 (b2)

mipip11 (b3)

mipip16 (b4)

mipip2@1

mipip7

mipip12

mipip17

mipip3 (b5)

mipip8 (b6)

mipip13 (b7)

mipip18 (b8)

mipip4 (b9)

mipip9 (b10)

mipip14 (b11)

mipip19 (b12)

mipip5 (b13)

mipip10 (b14)

mipip15 (b15)

mipip20 (b16);

C BY mipip1* (c1)

mipip6 (c2)

mipip11 (c3)

mipip16 (c4)

mipip2 (c5)

mipip7 (c6)

mipip12 (c7)

mipip17 (c8)

mipip3@1

mipip8

mipip13

mipip18

mipip4 (c9)

mipip9 (c10)

mipip14 (c11)

mipip19 (c12)

mipip5 (c13)

mipip10 (c14)

mipip15 (c15)

mipip20 (c16);

N BY mipip1* (d1)

mipip6 (d2)

mipip11 (d3)

mipip16 (d4)

mipip2 (d5)

mipip7 (d6)

mipip12 (d7)

mipip17 (d8)

mipip3 (d9)

mipip8 (d10)

mipip13 (d11)

mipip18 (d12)

mipip4@1

mipip9

mipip14

mipip19

mipip5 (d13)

mipip10 (d14)

mipip15 (d15)

mipip20 (d16);

O BY mipip1* (e1)

mipip6 (e2)

mipip11 (e3)

mipip16 (e4)

mipip2 (e5)

mipip7 (e6)

mipip12 (e7)

mipip17 (e8)

mipip3 (e9)

mipip8 (e10)

mipip13 (e11)

mipip18 (e12)

mipip4 (e13)

mipip9 (e14)

mipip14 (e15)

mipip19 (e16)

mipip5@1

mipip10

mipip15

mipip20;

MODEL PRIORS: a1-e16~N(0,.01); ! M=0, VAR=.01 small-variance priors on CL terms

OUTPUT: STDYX tech8;

**RC-CFA**

ANALYSIS: estimator=Bayes; fbiterations=10000; thin=10;

MODEL:

E BY mipip1

mipip6

mipip11

mipip16;

A BY mipip2

mipip7

mipip12

mipip17;

C BY mipip3

mipip8

mipip13

mipip18;

N BY mipip4

mipip9

mipip14

mipip19;

O BY mipip5

mipip10

mipip15

mipip20;

mipip1-mipip20 with mipip1-mipip20 (a1-a190); ! Labels residual covariances

mipip1-mipip20 (pload1-pload20); ! Labels residual variances

Model Priors: ! Sets model priors as Inverse Wishart with location and scale parameters as

! described in text and Appendix A

pload1~IW(275.6,400);

pload2~IW(194.4,400);

pload3~IW(376,400);

pload4~IW(300.8,400);

pload5~IW(280.4,400);

pload6~IW(331.2,400);

pload7~IW(339.6,400);

pload8~IW(434.4,400);

pload9~IW(370,400);

pload10~IW(327.6,400);

pload11~IW(327.2,400);

pload12~IW(345.6,400);

pload13~IW(322.4,400);

pload14~IW(295.2,400);

pload15~IW(294.4,400);

pload16~IW(259.6,400);

pload17~IW(217.6,400);

pload18~IW(286.8,400);

pload19~IW(437.6,400);

pload20~IW(220,400);

a1-a190~IW(0,400);

OUTPUT: STDYX tech8;

**AR-CFA W/ AVERAGES FOR SIMILAR EFFECTS**

! All AR-CFA code would be the same as above, except the AR effects about be labeled as:

rmipip2 on rmipip1;

rmipip3 on rmipip2;

rmipip4 on rmipip3;

rmipip5 on rmipip4;

rmipip6 on rmipip5 (oe1);

rmipip7 on rmipip6 (ea2);

rmipip8 on rmipip7 (ac2);

rmipip9 on rmipip8 (cn2);

rmipip10 on rmipip9 (no2);

rmipip11 on rmipip10 (oe2);

rmipip12 on rmipip11 (ea3);

rmipip13 on rmipip12 (ac3);

rmipip14 on rmipip13 (cn3);

rmipip15 on rmipip14 (no3);

rmipip16 on rmipip15 (oe3);

rmipip17 on rmipip16 (ea4);

rmipip18 on rmipip17 (ac4);

rmipip19 on rmipip18 (cn4);

rmipip20 on rmipip19 (no4);

! E terms

rmipip6 on rmipip1 (ee1);

rmipip11 on rmipip6 (ee2);

rmipip16 on rmipip11 (ee3);

! A terms

rmipip7 on rmipip2 (aa1);

rmipip12 on rmipip7 (aa2);

rmipip17 on rmipip12 (aa3);

! C terms

rmipip8 on rmipip3 (cc1);

rmipip13 on rmipip8 (cc2);

rmipip18 on rmipip13 (cc3);

! N terms

rmipip9 on rmipip4 (nn1);

rmipip14 on rmipip9 (nn2);

rmipip19 on rmipip14 (nn3);

! O terms

rmipip10 on rmipip5 (oo1);

rmipip15 on rmipip10 (oo2);

rmipip20 on rmipip15 (oo3);

MODEL CONSTRAINT:

! Now the labels are used to construct averages for hypothesis testing

NEW (ea ac cn no oe ee aa cc nn oo); ! These will be the averages for each type of effect

ea = (ea2+ea3+ea4)/3;

ac = (ac2+ac3+ac4)/3;

cn = (cn2+cn3+cn4)/3;

no = (no2+no3+no4)/3;

oe = (oe1+oe2+oe3)/3;

ee = (ee1+ee2+ee3)/3;

aa = (aa1+aa2+aa3)/3;

cc = (cc1+cc2+cc3)/3;

nn = (nn1+nn2+nn3)/3;

oo = (oo1+oo2+oo3)/3;

! Model output will now contain these parameters as estimates

OUTPUT: STDYX tech8;

**AR-CFA W/ MODEL CONSTRAINTS FOR SIMILAR AR EFFECTS (as per the previous model input, except instituting constraints rather than computing averages)**

! All input is the same as per the regular AR-CFA, except the AR terms are as follows:

rmipip2 on rmipip1;

rmipip3 on rmipip2;

rmipip4 on rmipip3;

rmipip5 on rmipip4;

rmipip6 on rmipip5 (oe1); ! Each label will be repeated in order to institute each constraint

rmipip7 on rmipip6 (ea1);

rmipip8 on rmipip7 (ac1);

rmipip9 on rmipip8 (cn1);

rmipip10 on rmipip9 (no1);

rmipip11 on rmipip10 (oe1); ! The same label as before, constraining AR effects to equality

rmipip12 on rmipip11 (ea1);

rmipip13 on rmipip12 (ac1);

rmipip14 on rmipip13 (cn1);

rmipip15 on rmipip14 (no1);

rmipip16 on rmipip15 (oe1); ! Same as previous

rmipip17 on rmipip16 (ea1);

rmipip18 on rmipip17 (ac1);

rmipip19 on rmipip18 (cn1);

rmipip20 on rmipip19 (no1);

! E terms

rmipip6 on rmipip1 (ee1); ! All of similar effects here are constrained to equality via labels

rmipip11 on rmipip6 (ee1);

rmipip16 on rmipip11 (ee1);

! A terms

rmipip7 on rmipip2 (aa1);

rmipip12 on rmipip7 (aa1);

rmipip17 on rmipip2 (aa1);

! C terms

rmipip8 on rmipip3 (cc1);

rmipip13 on rmipip8 (cc1);

rmipip18 on rmipip13 (cc1);

! N terms

rmipip9 on rmipip4 (nn1);

rmipip14 on rmipip9 (nn1);

rmipip19 on rmipip14 (nn1);

! O terms

rmipip10 on rmipip5 (oo1);

rmipip15 on rmipip10 (oo1);

rmipip20 on rmipip15 (oo1);

MODEL CONSTRAINT:

! Now we create a new parameter for each kind of AR effect

! This is not actually necessary for the constraints, which are done via the labeling above

! However, Mplus will give clear output for each of these new parameters, which makes

! interpreting model output slightly easier (this is a personal preference by the authors)

NEW (ea ac cn no oe ee aa cc nn oo);

ea1 = ea;

ac1 = ac;

cn1 = cn;

no1 = no;

oe1 = oe;

ee1 = ee;

aa1 = aa;

cc1 = cc;

nn1 = nn;

oo1 = oo;

**AR-CFA W/ SEPARATE CONSTRAINTS FOR normal🡪normal and reverse🡪reverse scoring; and normal🡪reverse and reverse🡪normal scoring**

! All input would be the same as the original AR-CFA, except for the following constraints

rmipip2 on rmipip1;

rmipip3 on rmipip2;

rmipip4 on rmipip3;

rmipip5 on rmipip4;

rmipip6 on rmipip5 (oe_c1); ! This effect is labeled c1 to reflect the ‘crossed’ nature of the

! AR effect among a normally-scored and a reverse-scored item

rmipip7 on rmipip6 (ea_s1); ! This effect is labeled s1 to reflect the ‘similar’ nature of the

! AR effect among either two normally-scored items or two reverse-scored items

rmipip8 on rmipip7 (ac_s1);

rmipip9 on rmipip8 (cn_s1);

rmipip10 on rmipip9 (no_s1);

rmipip11 on rmipip10 (oe_c1);

rmipip12 on rmipip11 (ea_s1);

rmipip13 on rmipip12 (ac_s1);

rmipip14 on rmipip13 (cn_s1);

rmipip15 on rmipip14 (no_c1);

rmipip16 on rmipip15 (oe_s1);

rmipip17 on rmipip16 (ea_s1);

rmipip18 on rmipip17 (ac_s1);

rmipip19 on rmipip18 (cn_s1);

rmipip20 on rmipip19 (no_s1);

! E terms

rmipip6 on rmipip1 (ee_c1);

rmipip11 on rmipip6 (ee_c1);

rmipip16 on rmipip11 (ee_c1);

! A terms

rmipip7 on rmipip2 (aa_c1);

rmipip12 on rmipip7 (aa_c1);

rmipip17 on rmipip2 (aa_c1);

! C terms

rmipip8 on rmipip3 (cc_c1);

rmipip13 on rmipip8 (cc_c1);

rmipip18 on rmipip13 (cc_c1);

! N terms

rmipip9 on rmipip4 (nn_c1);

rmipip14 on rmipip9 (nn_c1);

rmipip19 on rmipip14 (nn_c1);

! O terms

rmipip10 on rmipip5 (oo_c1);

rmipip15 on rmipip10 (oo_s1);

rmipip20 on rmipip15 (oo_s1);

MODEL CONSTRAINT:

! Again we do not need new parameters here as the constraints are instituted by labels above

! However, the output will contain the new parameters directly, which makes interpreting

! results easier (again, this is a personal preference only)

NEW (ea_s ac_s cn_s no_s no_c oe_c oe_s ee_c aa_c

cc_c nn_c oo_c oo_s);

! Recall that all effects ending in ‘s’ are similarly scored items, meaning normal-normal or

! reverse-reverse, whereas all effects ending in ‘c’ are differently scored items, meaning

! normal-reverse or reverse-normal. See text for details.

ea_s1= ea_s;

ac_s1= ac_s;

cn_s1= cn_s;

no_s1= no_s;

no_c1= no_c;

oe_c1= oe_c;

oe_s1= oe_s;

ee_c1= ee_c;

aa_c1= aa_c;

cc_c1= cc_c;

nn_c1= nn_c;

oo_c1= oo_c;

oo_s1= oo_s;

**AR-CFA W/ PLAUSIBLE VALUES GENERATED AS OUTPUT**

ANALYSIS: estimator=Bayes;

fbiterations=10000; thin=200; Processors=2; ! Allows 2,000,000 iterations

! Here the MODEL statement would be the same as the AR-CFA specified above

! At the conclusion of the input, however, the following would be included:

DATA IMPUTATION: ! Alerts Mplus to create data for missing/latent variables

NDATASETS = 20; ! The number of imputations/plausible values, in this case 20

SAVE = PV.*.dat; ! The name of the 20 files, which will all begin with PV

! The following command tells Mplus how and what to save

SAVEDATA: FILE = NA.dat; ! Irrelevant name of file that will save original data

SAVE = FSCORES (20); ! In combination with Bayes estimator and DATA IMPUTATION, ! this requests that 20 plausible values be generated

FACTORS = E A C N O; ! These are the latent factors for plausible value generation

Online Appendix B

Model Selection for a RC-CFA Model

We follow the guidance of Aspaurohov and Muthen (2015), who suggest sensitivity analyses that vary the degrees of freedom of distribution (*d*^[[1]](#footnote-1)^) to select the best RC-CFA. First, we start with selecting a *d* = 150 for our sample (*N* = 8,569) with an IC-CFA (χ^2^ =6052.87, *df* = 160). We then iterate five times for *d* = 25, 150, 400, 1500, and 5100 and find PPP = .50, .51, .27, 0, and 0, respectively. This approach keeps BSEM sensitivity analyses to five different models, searching for one that has a *d* value that yields fast convergence and PPP > .05. To cover a range of *d* values in a sample of 8,560, we examined five models that included *d* = 25 and *d* = 5100 on lower and upper ends, tracking speed of convergence and PPP. Results showed that a PPP > .05 value (PPP = .27), while also obtaining a fast convergence rate, with *d* above 400 showing PPP values below .05. Thus, our RC-CFA model has model priors with *d* = 400.

| *d* | Convergence Iteration  (PSR < 1.05) | DIC | pD | PPP > .05 |  |
| --- | --- | --- | --- | --- | --- |
| 25 | 11500 | 482285.63 | 195.67 | 0.495 |  |
| 150 | 1500 | 482221.48 | 221.76 | 0.506 |  |
| 400* | 500 | 482324.57 | 215.74 | 0.27 |  |
| 1500 | 200 | 482521.81 | 187.83 | 0 |  |
| 5100 | 100 | 483451.56 | 143.59 | 0 |  |

* According to the BSEM sensitivity analyses offered by Aspaurohov and Muthen (2015) BSEM, *d* = 400 is the best (not rejected) model with fastest convergence and a PPP > .05.

Online Appendix C

Example of Mplus Code for Examining Increasing/Decreasing AR Effects

MODEL:

! Here the typical AR-CFA specification would be included, except common terms might

! also include the following label and constraint specification

e2 on a2 (ea2); ! AR terms with parameter labels in parentheses

e3 on a3 (ea3);

e4 on a4 (ea4);

MODEL CONSTRAINT:

new(linear test); ! New linear parameter to impose a linear trend on the AR terms and test

! parameter to show the degree to which the linear term deviates from 1. This imposes a

! linear trend in AR effects while testing whether AR effects increase/decrease over time

ea1 = ea2*linear;

ea2 = ea3*linear;

ea3 = ea4*linear;

test = 1 - linear; ! If test > 0 then AR effects increase over time; if < 0 then they decrease

1. BSEM Residual Covariance analysis uses an inverse Wishart prior Θ ~ IW (*dD*, *d*), where Θ is the equivalent of diagonal matrix *D* of baseline CFA estimate, and *d* is the degrees of freedom of the distribution (Aspaurohov & Muthen, 2015). [↑](#footnote-ref-1)
