# Supplementary material for: Modeling Measurement as a Sequential Process: Autoregressive Confirmatory Factor Analysis (AR-CFA)
Source: Front Psychol. 2019 Sep 20;10:2108. doi: 10.3389/fpsyg.2019.02108 (PMC6763968; doi:10.3389/fpsyg.2019.02108)
Supplement: Supplementary file 3 [file Data_Sheet_2.ZIP › Online Appendix D/Online Appendix D.docx]

Monte Carlo Simulations

A simulation study with AR-CFA and IC-CFA models was conducted with varying sample sizes of 250, 500, and 1,000 as well as AR effects of 0, 0.1, 0.2, and 0.3 (when an AR effect = 0 in the population model this implies an IC-CFA, i.e., no AR effects are present). The factor loadings, residual variances and other population parameters were set by relying on typical standards of standardized loadings of .8 and factor correlations of .2 (Onde & Alvarado 2018; Hallquist & Wiley 2018; Muthen & Muthen 2002).

The results show that: 1) The AR-CFA analysis model does not show parameter bias or meaningful differences in fit using SRMR and RMSEA even when the data are from an IC-CFA population model (i.e., when AR values are zero in the population and the AR-CFA reduces to an IC-CFA), so even if strict IC-CFA assumptions are met the AR-CFA can be used; 2) the IC-CFA analysis model under a population AR-CFA model systematically over-estimates factor loadings and factor covariances, which is consistent with our real-data example wherein the AR-CFA shows better discriminant validity, but with small AR parameter values (e.g., .1) this problem is minimal for the IC-CFA; 3) the AR-CFA analysis model has convergence problems with samples of 250, particularly as AR parameter values decrease towards zero, which suggests samples of at least 400-500 to estimate the AR-CFA, or perhaps using a Bayes estimator rather than maximum likelihood; and 4) the IC-CFA analysis models shows poorer performance in terms of model fit as AR parameter values get larger, and these levels of fit are often found for IC-CFA analysis models in practice, suggesting that unmodeled AR effects may be the cause of some observed misfit in the literature when using the IC-CFA, which again suggests that researchers should experiment with the AR-CFA in practice. Bias and efficiency estimate calculations can be found in Simulation Results Final.xlsx file for each simulation run. All simulation files and results are presented in Online Appendix D folder as a .zip file for download.

| Table 1. Fit Indices for Simulation Results | | | | | | | | | |
| --- | --- | --- | --- | --- | --- | --- | --- | --- | --- |
| SAMPLE | AR EFFECT | ANALYZED BY | SUCCESFUL COMPUTATIONS | CHI-SQUARE (df) | CHI-SQUARE DIFFERENCE TESTING df=34 vales and significance ^(1)^ | RMSEA | SRMR | AVERAGE FACTOR LOADING BIAS | AVERAGE FACTOR COVARIANCE BIAS |
| 250 | 0 | AR-CFA | 653 | 125.57 (126) | 40.29 | 0.009 | 0.035 | -0.00081 | -0.02200 |
| 250 | 0 | IC-CFA | 1000 | 165.86 (160) |  | 0.012 | 0.036 | -0.00349 | -0.00185 |
| 250 | 0.1 | AR-CFA | 633 | 125.08 (126) | 86.08*** | 0.009 | 0.034 | 0.00152 | -0.03485 |
| 250 | 0.1 | IC-CFA | 1000 | 211.16 (160) |  | 0.035 | 0.036 | 0.01141 | 0.00250 |
| 250 | 0.2 | AR-CFA | 798 | 126.75 (126) | 213.78*** | 0.01 | 0.034 | 0.00391 | -0.01490 |
| 250 | 0.2 | IC-CFA | 1000 | 340.53 (160) |  | 0.067 | 0.039 | 0.03295 | 0.07180 |
| 250 | 0.3 | AR-CFA | 892 | 127.62 (126) | 405.52*** | 0.01 | 0.033 | 0.00095 | -0.00005 |
| 250 | 0.3 | IC-CFA | 1000 | 533.14 (160) |  | 0.096 | 0.045 | 0.05941 | 0.19430 |
|  |  |  |  |  |  |  |  |  |  |
| 500 | 0 | AR-CFA | 934 | 125.07 (126) | 36.92 | 0.006 | 0.024 | 0.00074 | -0.01435 |
| 500 | 0 | IC-CFA | 1000 | 161.99 (160) |  | 0.007 | 0.025 | -0.00096 | 0.00155 |
| 500 | 0.1 | AR-CFA | 888 | 125.09 (126) | 127.98*** | 0.006 | 0.024 | 0.00246 | -0.02490 |
| 500 | 0.1 | IC-CFA | 1000 | 253.07 |  | 0.034 | 0.027 | 0.01338 | 0.00478 |
| 500 | 0.2 | AR-CFA | 950 | 126.26 (126) | 385.45*** | 0.006 | 0.023 | 0.00309 | -0.00110 |
| 500 | 0.2 | IC-CFA | 1000 | 511.71 (160) |  | 0.066 | 0.031 | 0.03449 | 0.07320 |
| 500 | 0.3 | AR-CFA | 988 | 127.07 (126) | 769.9*** | 0.007 | 0.023 | 0.00065 | -0.00030 |
| 500 | 0.3 | IC-CFA | 1000 | 896.97 (160) |  | 0.096 | 0.038 | 0.06063 | 0.19275 |
|  |  |  |  |  |  |  |  |  |  |
| 1000 | 0 | AR-CFA | 998 | 125.4 (126) | 35.24 | 0.004 | 0.017 | 0.00023 | -0.00790 |
| 1000 | 0 | IC-CFA | 1000 | 160.64 (160) |  | 0.004 | 0.018 | -0.00053 | -0.00140 |
| 1000 | 0.1 | AR-CFA | 996 | 125.09 (126) | 216.31*** | 0.004 | 0.016 | 0.00134 | -0.01085 |
| 1000 | 0.1 | IC-CFA | 1000 | 341.40 (160) |  | 0.034 | 0.02 | 0.01475 | 0.01300 |
| 1000 | 0.2 | AR-CFA | 995 | 126.71 (126) | 731.57*** | 0.005 | 0.016 | 0.00169 | 0.00500 |
| 1000 | 0.2 | IC-CFA | 1000 | 858.28 (160) |  | 0.066 | 0.026 | 0.03520 | 0.07260 |
| 1000 | 0.3 | AR-CFA | 1000 | 127.02 (126) | 1502.31*** | 0.005 | 0.016 | 0.00051 | 0.00315 |
| 1000 | 0.3 | IC-CFA | 1000 | 1629.33 (160) |  | 0.096 | 0.034 | 0.06162 | 0.19395 |
|  |  |  |  |  |  |  |  |  |  |
|  |  |  |  |  |  |  |  |  |  |
|  |  |  |  |  |  |  |  |  |  |
| (^1^) p = .05*, chi-sq difference for (df = 34) 48.602 | | | |  |  |  |  |  |  |
| (^1^) p = .01**, chi-sq difference for (df = 34) 56.061 | | | |  |  |  |  |  |  |
| (^1^) p = .001***, chi-sq difference for (df = 34) 65.247 | | | |  |  |  |  |  |  |
